# Global drivers of food system (un)sustainability: A multi-country correlation analysis

**DOI:** 10.1371/journal.pone.0231071

**Published:** 2020-04-03

**Authors:** Christophe Béné, Jessica Fanzo, Steven D. Prager, Harold A. Achicanoy, Brendan R. Mapes, Patricia Alvarez Toro, Camila Bonilla Cedrez

**Affiliations:** 1 Decision and Policy Analysis Program, International Center for Tropical Agriculture, Cali, Colombia; 2 School of Advanced International Studies, Johns Hopkins University, Washington, DC, United States of America; 3 Frederick S. Pardee Center for International Futures, University of Denver, Denver, CO, United States of America; 4 Department of Environmental Science and Policy, University of California, Davis, CA, United States of America; SOAS, University of London, UNITED KINGDOM

## Abstract

At present, our ability to comprehend the dynamics of food systems and the consequences of their rapid ‘transformations’ is limited. In this paper, we propose to address this gap by exploring the interactions between the sustainability of food systems and a set of key drivers at the global scale. For this we compile a metric of 12 key drivers of food system from a globally-representative set of low, middle, and high-income countries and analyze the relationships between these drivers and a composite index that integrates the four key dimensions of food system sustainability, namely: food security & nutrition, environment, social, and economic dimensions. The two metrics highlight the important data gap that characterizes national systems’ statistics—in particular in relation to transformation, transport, retail and distribution. Spearman correlations and Principal Component Analysis are then used to explore associations between levels of sustainability and drivers. With the exception of one economic driver (trade flows in merchandise and services), the majority of the statistically significant correlations found between food system sustainability and drivers appear to be negative. The fact that most of these negative drivers are closely related to the global demographic transition that is currently affecting the world population highlights the magnitude of the challenges ahead. This analysis is the first one that provides quantitative evidence at the global scale about correlations between the four dimensions of sustainability of our food systems and specific drivers.

## Introduction

Within the complex agenda on food security and nutrition, food systems are being progressively recognized as a critical entry point for action [1–4]. Yet, our understanding about food system dynamics is still in a development stage [5]. While conceptual and theoretical progress has been made around definitions, indicators, and metrics describing food systems [6–8], researchers and analysts are still struggling with a core element of the problem: How (un)sustainable our food systems are and what drives this (un)sustainability?

Part of our collective inability to adequately understand food systems derives from the fragmented and static datasets that are available in relation to food systems. This limits our ability to comprehend holistically the dynamics and complexity of those systems, to investigate properly how they evolve, what drives them, and to anticipate the consequences of their rapid evolutions. The implications are broad and not only affect nutrition and human health, but also the environment, e.g., [4], and the social and economic wellbeing of people [3,9].

At present, more than 820 million people remain undernourished [10], 149 million children are stunted, and 49.5 million are wasted [11]. While the precise figures are uncertain, there are an estimated 2 billion people with micronutrient deficiencies and 2.1 billion adults overweight or obese [12]. Overall, “unhealthy” diets are estimated to be the most significant risk factor for global burden of disease in the world [2]. Simultaneously, food systems are recognized to be one of the largest causes of global environmental changes [13], leading to soil degradation, deforestation, and depletion of freshwater resources [14–16]. Recent estimates show that food production is responsible for 19 to 29% of global greenhouse gas emissions [17].

Taken together, such figures and statistics highlight the urgent need to improve our understanding of the *dynamics* of food systems. We must assess not only the degree to which these dynamics are (or are not) sustainable [4, 15] but also the nature of the *main drivers* that affect them. Understanding the state of food system sustainability in relation to the drivers that affect this sustainability is indeed critical if we want to support policy-makers in designing and implementing adequate policy and interventions.

The objective of this paper is to address these gaps by exploring more thoroughly the interactions between the sustainability of food systems and key drivers at a global scale. For this, we use two independent metrics: one on food systems’ sustainability (based on the sustainability map recently proposed by [18]), and one on food systems’ drivers, using indicators available for a common set of countries from low, middle, and high-income levels. Spearman correlations and Principal Component Analysis are then used to explore the relations between levels of (un)sustainability and drivers across the set of countries. The analysis reveals that with the exception of changes in trade flows of merchandise and services, all the other moderate to strong associations between key drivers and food system sustainability are negative. This finding is consistent with the current observation that, at present, “food systems are failing us” [3, p. 17]. While those results are not totally unexpected, this analysis is the first one that is able to provide quantitative evidence at a global scale of the existence of significant correlations between the unsustainability of our food systems and specific drivers.

## Methods

The analysis is based on two independent metrics that were constructed for a common set of low, middle, and high-income countries; one on food systems’ sustainability, and one on food systems’ drivers.

### Food system sustainability metric

The protocol used to build the metric of food system sustainability has been developed and presented in detail in [18]. What follows below is a brief technical overview of how the metric and the country’s associated sustainability scores were built. The first step was a thorough literature review that was used to identify the relevant peer-reviewed articles, documents, and reports published in English between 2000 and 2018 that discuss indicators and metrics of food system sustainability. The search was conducted using multidisciplinary databases including Google scholar, JSTOR, and Scopus, and searching for the keywords “food system(s)” AND (“sustainability” OR “sustainable”) in the title or abstract of the documents. After removing of duplicates, 83 documents were retained (see references in S1 Table for the complete list). The review of those 83 document indicated that four dimensions of sustainability are generally acknowledged in the literature related to food systems: environment, economic, social and food security & nutrition. The food system indicators mentioned in the 83 documents were then systematically recorded. One hundred and ninety-two different indicators were identified and documented, along with the dimensions, components and, when available, domains of the metric to which those indicators were linked.

The list of 192 indicators was refined using nine inclusion/exclusion criteria specifically considered to address methodological and conceptual issues revealed by the literature review–see [18] for details. Exclusion criteria included: evidence of cross-correlation between indicators, use of latent variables, composite, and non-comparability nature of the indicators. Inclusion criteria included: conceptual relevance, global scale, global validity, time period (2000–2018), and clear methodology. The detailed definitions of those inclusion/exclusion criteria and the issues they addressed are provided in S2 Table. Applying the inclusion/exclusion criteria to the pool of 192 indicators resulted in a short-list of 27 indicators (see diagram in S1 Fig for a PRISMA flow diagram of the selection process).

The next step was to compute countries’ sustainability scores. Indicators were first normalized using Box-Cox transformation to avoid issues associated with potential heteroskedastic datasets, and then normalized using a standard min-max transformation with a [0, 1] range. Based on the literature on composite indicators and multi-criteria analysis (see e.g. [19–21]), a formula was developed for the computation of the sustainability score. Two criteria were considered for this: (i) the potential degree of compensation that exists between the four dimensions of the metric; and (ii) the cross-correlations between indicators within each dimensions of the score. A minimal level of compensation was assumed between the four dimensions of the metric (which means that no particular dimension of the sustainability metric was expected to be fully substitutable by any other). Based on this reasoning and the results of the cross-correlation matrixes estimated for each of those dimensions (see diagram in S2 Fig), the sustainability score formula was:

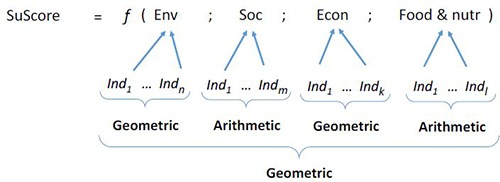
(1)
where SuScore is the country sustainability score, *Ind*_*1*_ … *Ind*_*n*_, *Ind*_*m*_, *Ind*_*k*_, and *Ind*_*l*_ represent the groups of indicators associated with the four dimensions of the metric (Env, Soc, Econ, and Food&Nutr). ‘Geometric’ and ‘Arithmetic’ indicate the types of mean used within and between the different elements of the metric to aggregate the indicators.

One critical issue—albeit rarely discussed in papers dealing with the construction of global metrics—is that it is not possible to maximize both dimensions of the metric at the same time: a trade-off exists between the number of countries that can be included in the metric and the number of indicators used to build that metric; the larger the numbers of indicators, the lower the number of countries for which all indicators are available, and vice versa (see diagram in S3 Fig for an illustration of that trade-off). A three-criterion decision approach is then used to identify what combination (countries–indicators) is optimal, given the current availability of data. The three criteria were: (i) the number of countries that are dropped out of the metric when the number of indicators is increased by one; (ii) the standard deviation of the aggregated mean of the countries’ scores when the number of indicators included in the metric is increased or decreased by one; and (iii) the degree to which countries’ ranks shift when a new indicator is included in the metric–see [18] for details. Using a simple minimal function approach, [18] was able to determine that the combination of 20 indicators– 97 countries is the optimal combination at present given the current datasets available.

### Food system drivers

The second step in the analysis is the identification of food system drivers. For this, peer-reviewed articles and gray literature discussing food systems were scanned, with the objective to identify which amongst those documents also treated the question of food system *drivers*. The keywords used for this search were “food system(s)” AND “driver(s)” in the title, abstract, or main body of the document (we acknowledge here that the term “driver(s)” is not (yet) systematically used in the food system literature and that its use as keyword may have led to the missing of a few potentially relevant articles). We used the same reference databases used by [18] for the sustainability indicators above (Google Scholar, JSTOR, and Scopus) and the search was limited to the same time period (2000–2018) and to English (language) literature. Thirty-three documents were identified after elimination of duplicates.

An inductive framework [22] was then used to determine whether a core set of drivers could be identified from these 33 documents. For this, we started from the definition of food system drivers proposed by [5] (drivers of food systems are “endogenous or exogenous processes that deliberately or unintentionally affect or influence a food system over a long-enough period of time so that their impacts result in altering durably the activities [or actors’ behaviors], and subsequently the outcomes, of that system” p.152 –modified by us). We then applied the inductive framework: statements made in the 33 documents on what processes influence food system changes were systematically recorded. Convergences and similarities in those statements were then established and a series of propositions on what driver(s) were generating these patterns, were produced. This process allowed us to isolate a subset of archetypal (or key) drivers.

For each of these key drivers, potential links with indicators (of the magnitude of change associated with drivers) were identified from publicly available datasets. In addition to their conceptual relevance, two additional criteria were used to determine the suitability of the indicators as drivers of food systems: (i) their global coverage (i.e. covering low-, middle- and high-income countries); and (ii) their availability for a period of time long enough for potential changes related to the cumulative and/or lagged impact of these drivers to be observable and estimated. This last criterion stems from the premise that what constitute the driving elements for most of the transitions or transformations that are observed in food systems are not just the results of the current (or past) levels of the process considered but the *change* in those levels over time. For instance, it is not just the temperature or the intensity of precipitation that is thought to lead to adaptations (or transformations) amongst the actors of food systems, but the *change* in temperature or intensity of precipitation relative to past conditions [17]. Likewise it is not the level of consumer income *per se* that is said to drive the rise in demand for animal-based protein as currently observed in middle income countries, but the relative *increase* in that consumer income [23,24]. An 8 to 15-year window (depending on the availability of the data) was therefore used to compute those changes in levels.

### Correlation between drivers and level of sustainability

The correlations between individual drivers and sustainability scores were established by computing Spearman correlation coefficients (*ρ)* between the two country-level series after outliers were removed (using the R package OutliersO3). Spearman correlation was chosen because it does not require continuous-level data (as it uses ranks) and does not assume normal distribution of the variables; as such it is more robust than the Pearson correlation test [25].

The statistical significance of the correlation coefficients (*ρ)* were computed at *α* = 0.05. The analysis allowed us to test the null hypothesis *H*_*o*_ (namely that there is no monotonic relation between drivers and sustainability scores). In this context, a *p* ≥ 0.05 would signify that *Ho* could not be rejected, i.e. that no significant correlation exists. In parallel, the strength of the correlation coefficients *ρ* was assessed using a 5-scale system as proposed by [26] where a case of 0.0 ≤ I*ρ*I < 0.2 indicates a very weak correlation; 0.2 ≤ I*ρ*I < 0.4 = weak; 0.4 ≤ I*ρ*I < 0.6 = moderate; 0.6 ≤ I*ρ*I < 0.8 = strong; 0.8 ≤ I*ρ*I ≤ 1.0 = very strong correlation. Note that the two tests are independent in the sense that the level of statistical significance of the Spearman correlation does not bear any implication about the strength of the relationship (in that a relationship can be strongly significant but weak–or vice versa).

### Principal component analysis

While a one-to-one correlation analysis between the food sustainability score and drivers provides a solid representation of the dynamics between the two factors taken individually (although the directionality of the link may not be obvious), it does not control for potential confounding factors and/or multicollinearity between drivers. Multivariable analyses would be necessary to address those issues. However, because the set of indicators used to proxy the drivers varies from one country to the next, running a multi-variable correlation analysis with the 20 drivers as explanatory variables would be possible only with 37 countries–which would significantly reduce the robustness and the representativeness of the analysis. Instead we performed a Principal Component Analysis (PCA). The motivation was to go beyond the one-to-one basis of the Spearman correlation and to consider all drivers together in order to identify potential interactions between those drivers. We used the ‘PCA’ command from FactoMineR library in R^®^ to perform the analysis, and the model is computed using the correlation matrix of the variables (drivers).

## Results

Understanding the dynamics of food system sustainability requires unpacking the relationships between indicators of sustainability and the factors that are deemed most relevant in terms of their influence thereon. This influence results from a combination of both direct and indirect association between drivers and indicators and, as such, necessitates exploration first of the indicators, then the drivers, and finally their associations.

### Assessing the sustainability of food systems

The first component of our analysis was therefore the assessment of the sustainability of food systems. For this, we replicated the approach developed and presented in their recent paper by [18]. Four dimensions of sustainability were included in the analysis to capture food system’s sustainability: environmental, economic, social, and food security & nutrition dimensions [2,27–29]. All four dimensions are complex and compound in nature, and two of them (the environmental and food security and nutrition dimensions) are usually further decomposed into components. For environment, the most frequently discussed components are: air, water, soils and land, (bio)diversity, and energy [6,30–32], and for food and nutrition, those components are: food security, food safety, food waste and losses, and nutrition [33–35].

Following the protocol designed by [18], the four dimensions and their corresponding components were combined to represent the first two levels of the food system sustainability metric. Specific domains were then added to each dimension. For food security, the four domains conventionally referred to in the literature are: availability, accessibility, utilization, and stability [36,37]. For water, and soils and land, two domains are usually considered: ‘quality’ and ‘use’. Those were therefore incorporated in the metric, while for biodiversity, wildlife and agrobiodiversity were included. For the social dimension, the literature indicates that issues of equity, gender and inclusion are commonly considered [38,39]. Less consensus exists regarding the representation of economic sustainability. Financial performance (creation of value added), employment rate, and economic inequality were selected for this study [31,35]. For the nutrition component, diet and the triple burden of malnutrition (undernutrition, overweight and obesity, and nutrient deficiency) were included [37,40,41]. In total, this results in 27 domains covering the four dimensions of food system sustainability (see Table 1). For each of these 27 domains, available indicators were compiled from publicly available databases (e.g. World Bank, IMF, UN-agencies)–see fourth column in Table 1.

**Table 1:** The four dimensions, 27 domains and associated indicators used to build the metric of food system sustainability. The 20 indicators indicated with an * are those which were eventually retained by [18] to calculate the 97 countries’ sustainability scores.

Dimensions	Components	Domains	Indicators^(1)^	Nber of countries^(2)^
**Environment**	Air	■ Quality	Greenhouse gas emission*	222
	Water	■ Quality	*Water pH*	*74*
		■ Use	Agricultural water withdrawal*	174
	Soils and land	■ Quality	Soil carbon content*	202
		■ Use	Agricultural land as % of arable land*	217
	Biodiversity	■ Wildlife (plants, animals)	Benefits of biodiversity index*	192
		■ Crop diversity	Crop diversity index*	177
	Energy	■ Use	*Agriculture and forestry energy used as a percentage of total energy use*	*113*
**Economic**		■ Financial performance	Agriculture value-added per worker*	181
		■ Employment rate	*Agriculture under-employment (%)*	*72*
		■ Economic distribution	*Gini index for land distribution*	*86*
**Social**		■ Gender / Equity	Labor force participation rate, female*	184
		■ Inclusion (international)	*Predominance of fair trade organizations and producers*	*160*
		■ Inclusion (national)	*Employment in agriculture*	*149*
**Food & Nutrition**	Food Security	■ Availability	Per capita food available for human consumption*	113
		■ Access (affordability)	Food consumption as share of total income*	113
		■ (Physical) accessibility	Estimated travel time to the nearest city of 50,000 or more people*	245
		■ Utilization – water	Access to improved water resource*	198
		■ Utilization – energy	Access to electricity*	211
		■ Stability (economic)	Price volatility index*	194
		■ Stability (supply)	Per capita food supply variability*	162
	Food Safety	■ Safety	Burden of foodborne illness*	194
	Food Waste and Use	■ Loss and waste	Food loss as % of total food produced*	113
	Nutrition	■ Diet	Diet diversification*	165
		■ Undernutrition	*Stunting*	*129*
		■ Overnutrition	Prevalence of obesity*	191
		■ Nutrient deficiency	Vitamin A deficiency*	193

Derived from [18].

Notes: ^(1)^ Detailed definition of the 27 indicators are provided in S3 Table.

^(2)^ Number of countries based on the ISO standard “country code” list, which includes 249 countries, territories and areas of geographical interest.

Cursory inspection of the existing databases reveals that the same indicators are rarely available across the full range of low, middle, and high-income countries. In other instances, indicators are available, but only for a sub-set of countries that lack coverage with the rest of the indicators. As a result, given the *current availability of data at the global level*, it is not possible to build a full metric of food system sustainability including all countries in the world. A compromise needs to be made between the number of countries and the number of indicators to be included in the metric. Based on the decision criteria presented in the methodology section, [18] show that the optimal combination that balances number of countries and dimensionality of representation is 20 indicators and 97 countries.

**Fig 1:**
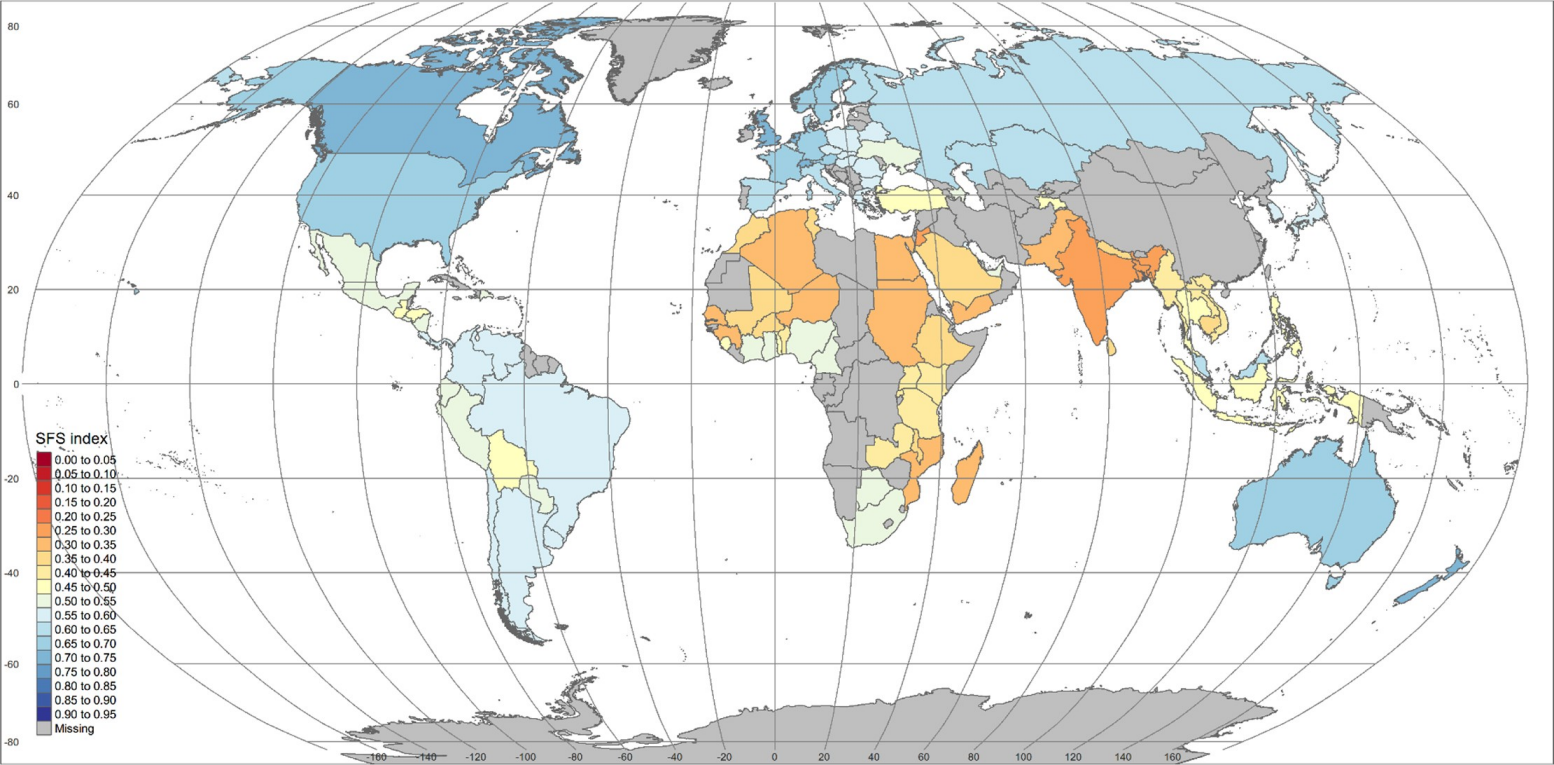
Food system sustainability score calculated for 97 countries and 20 indicators covering the four dimensions of food system: Environment, economic, social, and food security & nutrition. The list of indicators used to build the map is provided in Table 1. Country individual scores are provided in S4 Table. Reprinted from [18] under a CC BY license, with permission from *Scientific Data*, original copyright 2019.

Using formula (1) we then computed individual country’s sustainability scores for the 97 countries for which the 20 indicators were available. The resulting map is displayed in Fig 1. It is similar to the one presented in [18]. The proportions of low, middle, and high-income countries amongst those 97 countries are respectively 18% (L), 51% (M), and 31% (H), which is remarkably close to the proportions observed for the 218 countries and other regions listed in the World Bank 2017 list, respectively: 14% (L); 49% (M); 37% (H). The subsample of 97 countries included in this analysis is therefore a good representation (in terms of proportions of low, middle and high-income countries) of the 2017 situation in the world.

### Drivers of food system sustainability

The second step in the analysis consisted in integrating the major drivers of food systems changes. Drawing on the review of food system drivers conducted by [5] and the methods presented earlier, 12 key drivers were identified from existing available datasets. Those 12 drivers are listed in the second column of Table 2, organized according to their contribution to the three major elements of food system: production-supply, trade-distribution, and demand-consumption.

**Table 2:** Food system drivers and their indicators.

Category	Drivers	Indicators ^(1)^	Database	Period	Selected period and method of calculation	Nber of countries
Demand/ Consumer	Population demographic transition	Change over time in population (%)	World Bank	1960–2016	median value over 2004–2016	214
	Raise in consumers' income	Change over time in GDP per capita (%)	World Bank	2000–2016	median value over 2004–2016	192
	Urbanization and associated changes in life style	Change over time in urban population (% of total)	World Bank	1960–2016	difference in medians between 2004/2006 and -2014/2016	213
		Change over time in female employment in services (%)	World Bank	1963–2016	difference in medians between 2004/2006 and -2014/2016	92
	Growing attention paid to diet	Change over time in interest in healthy diet	Google Trends^(2)^	2004–2018	linear slope estimation (Sen method)^(4)^	68
Production/ Supply	Technological Innovation	Change over time in ratio of cereal crop yield and fertilizer application	World Bank	2002–2014	difference in medians between 2001/2003 and 2012/2014	149
		Change over time in fertilizer use (kg/ha of arable land)	World Bank	2002–2014	difference in medians between 2001/2003 and 2013/2015	152
	Intensification of the agricultural sector	Change over time in cereal yield (kg per hectare)	World Bank	1961–2016	difference in medians between 2004/2006 and 2014/2016	179
		Change over time in agricultural area (% of land area)	World Bank	1961–2015	difference in medians between 2003/2005 and 2014/2016	206
	Improved access to infrastructure	Change in access to electrical grid	DMSP OLS^(3)^	2008–2015	difference in values between 2008 and 2015	246
	General degradation in agro-ecological conditions	Land degradation (GLASOD degrees)	FAOSTAT data	1991	1991^(5)^	180
		Soil erosion (GLASOD degrees)	FAOSTAT data	1991	1991^(5)^	143
	Climate change	Change over time in mean temperature (degree Celsius)	World Bank	1991–2015	linear slope estimation^(4)^ on annual temperature values	227
		Change over time in total precipitation (mm)	World Bank	1991–2015	linear slope estimation^(4)^ on annual precipitation values	205
		Change over time in temperature variability (degree Celsius)	World Bank	1991–2015	linear slope estimation^(4)^ on increase in annuals standard deviations	227
		Change over time in precipitation variability (mm)	World Bank	1991–2015	linear slope estimation^(4)^ on increase in annual standard deviations	205
Trade/ Distribution	Policies facilitating / mitigating trade	Change over time in food exports (% of merchandise exports)	World Bank	2000–2015	difference in medians between 2003/2005 and 2014/2016	144
	Internationalization of private investments	Change over time in foreign direct investment (US$ per capita)	World Bank	2000–2015	difference in medians between 2003/2005 and 2014/2016	196
		Change over time in merchandise and services trade (US$ per capita)	World Bank	2000–2015	difference in medians between 2003/2005 and 2014/2016	197
	Growing concerns for food safety	Change over time in concerns in food safety issues	Google Trends	2004–2019	linear slope estimation^(4)^	69

^(1)^ Detailed definitions of the drivers‘ indicators are provided in data in S5 Table

^(2)^ Google trends: see details in S5 Table

^(3)^ DMSP OLS: Global Radiance-Calibrated Nighttime Lights Version 4, Defense Meteorological Program Operational Linescan System), time difference own calculation

^(4)^ Sen’s method [109]

^(5)^ for two datasets (Land degradation and Soil erosion), only one year data are available (1991). For those two indicators, the driver effect was approximated by using the 1991 value (and not a change in value, unlike all the other drivers).

On the consumption/demand side, three distinct but related drivers are widely acknowledged in the literature: population growth, increases in (consumers’) income, and urbanization [22,30,42–43]. A fourth key driver that is expected to have increasing influence on the consumer/demand component in the future is the growing attention paid by the different actors of the food systems to diet and health-related issues [44]. Although only marginal influence is seen to date (see e.g. [45–47] for some examples), attention paid to diet and health-related issues is expected to become one of the key drivers on the consumption/demand side in the future.

On the production/supply side, several forms of technological innovation (agriculture mechanization, irrigation, plant breeding, etc.) along with intensification and homogenization of the agricultural sector have been identified as two major drivers of food systems changes [48–50]. Conjointly, improved access to local infrastructure (e.g. power grid, roads) and to urban centers [51–53] as well as to local supermarkets [54,55] have led to significant changes, especially for the smaller and medium-size producers in low and middle income countries (LMICs) [56–58]. In parallel, general degradation of soil quality and other agro-ecosystem conditions is widely recognized as a key driver of decrease in agricultural production [13,14,30]. The literature also identifies increases in temperature combined with higher intensity and severity of extreme weather events (which all fall under the generic category of “climate change”) as an important driver of food systems [3,59–63].

At the intersection of supply/production and distribution/trade, policies and other related national and international processes established to facilitate or mitigate trade expansion and exportation of agricultural products (e.g. subsidies in OECD countries) are another significant driver of food system changes [64–71]. On this distribution-trade side, the two other key drivers often mentioned in the literature are: internationalization of private investments (leading to, amongst others, the “supermarketization” of food systems [72]); and the growing concern from local and national policy makers/governments for food safety [73–75].

Once the drivers were identified, we looked for indicators or proxies for each of them in publicly available datasets. Twenty indicators were identified (meaning that for some drivers, more than one indicator/proxy could be identified). Those are listed in the third column of Table 2, along with the databases from which they were extracted, the time period and the number of countries for which they are available, and the selected periods and methods used to compute the changes in those indicators.

### Correlating levels of sustainability and drivers

Once the indicators of each key driver were identified, individual Spearman rank correlations (*ρ*) were estimated to evaluate the strength of the relationships between the drivers and the 97 countries’ sustainability scores. The results of the Spearman tests are presented in Table 3. They indicate that amongst the 12 drivers, three of them show moderate (0.4 ≤ I*ρ*I < 0.6) to strong (0.6 ≤ I*ρ*I < 0.8) levels of correlation with the sustainability scores, and seven show weak correlation (0.2 ≤ I*ρ*I < 0.4). No very strong correlation (I*ρ*I of 0.8 or greater) were observed. The statistical significance of the Spearman coefficients (determined for *α* = 0.05) indicates that the existence of those correlations is confirmed in nine out of the 20 correlations tested (Table 3).

**Table 3:** Spearman correlation tests between food system drivers and country food system sustainability scores. Results ranked from the strongest to the weakest coefficients *ρ* (in absolute values). Drivers above the dotted line are those displayed in Fig 2.

Drivers	Indicators	Spearman coef^(1)^	Strength^(2)^
Internationalization of private investments	Change over time in merchandise and services trade (US$ per capita)	0.701***	strong
Population demographic transition	Change over time in population (%)	-0.664***	strong
Intensification of the agricultural sector	Change over time in agricultural area (% of land area)	-0.471***	moderate
Urbanization and associated changes in life style	Change over time in female employment in services (% of female employment)	-0.343**	weak
Urbanization and associated changes in life style	Change over time in urban population (% of total)	-0.332***	weak
Technological Innovation	Change over time in fertilizer use (kg/ha of arable land)	-0.274**	weak
Intensification of the agricultural sector	Change over time in cereal yield (kg per hectare)	0.259**	weak
Internationalization of private investments	Change over time in foreign direct investment (US$ per capita)	0.220*	weak
Raise in consumers' income	Change over time in GDP per capita (%)	-0.212*	weak
Policies facilitating or mitigating trade	Change over time in food exports (% of merchandise exports)	0.211 ^NS^	weak
Climate change (extreme events)	Change over time in precipitation variability (mm)	-0.185 ^NS^	very weak
Technological Innovation	Change over time in ratio of cereal crop yield and fertilizer application	-0.163 ^NS^	very weak
Climate change (trend)	Change over time in mean temperature (degree Celsius)	0.137 ^NS^	very weak
General degradation in agro-ecological conditions	Soil erosion (GLASOD degrees)	-0.129 ^NS^	very weak
Improved access to infrastructure and information	Change in access to infrastructure and information (electrical change)	0.093 ^NS^	very weak
Climate change (extreme events)	Change over time in temperature variability (degree Celsius)	0.074 ^NS^	very weak
Growing concerns for food safety	Change over time in concerns in food safety issues	-0.064 ^NS^	very weak
Climate change (trend)	Change over time in total precipitation (mm)	-0.063 ^NS^	very weak
General degradation in agro-ecological conditions	Land degradation (GLASOD degrees)	0.015 ^NS^	very weak
Growing attention paid to diet and health	Change over time in interest in healthy diet	-0.003 ^NS^	very weak

^(1)^ Statistical significance of Spearman rank coefficients

*** = *p* < 0.001

** = *p* < 0.01

* = *p* < 0.5

^NS^ = non-significant. Null hypothesis H_o_: no monotonic relation between drivers and sustainability scores.

^(2)^ Strength of Spearman rank correlation (based on Myers and Well’s score system [26]): 0.0 ≤ I*ρ*I < 0.2 = very weak; 0.2 ≤ I*ρ*I < 0.4 = weak; 0.4 ≤ I*ρ*I < 0.6 = moderate; 0.6 ≤ I*ρ*I < 0.8 = strong; 0.8 ≤ I*ρ*I ≤ 1.0 = very strong.

**Fig 2:**
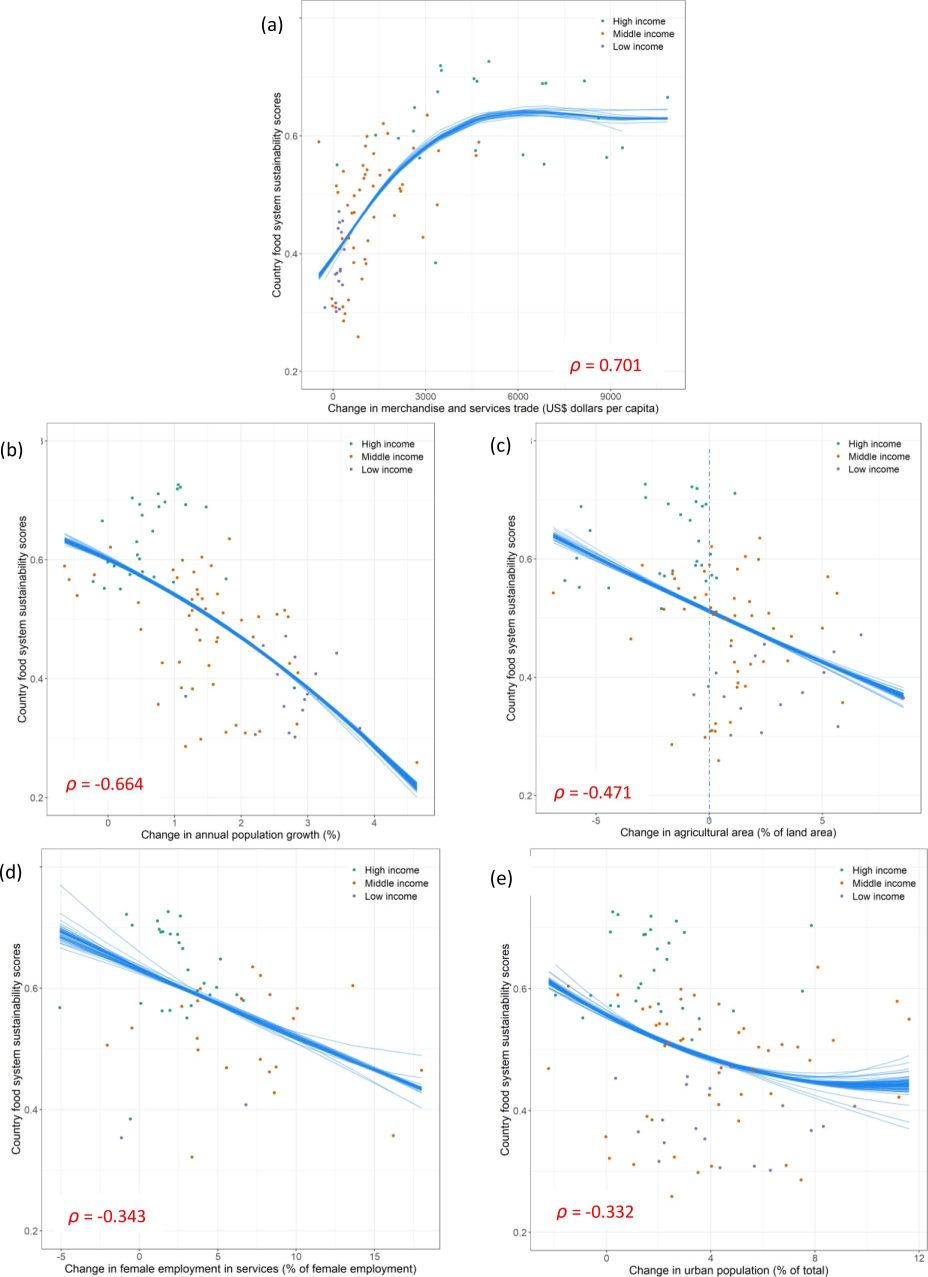
Relationship between country food system sustainability scores and key drivers. Only drivers displaying a Spearman correlation coefficient (*ρ)* greater than 0.3 are shown: (a) change in merchandise and services trade; (b) change in population growth; (c) changes in agricultural area; (d) change in women employment in services; and (e) change in urban population. Trends (blue lines) estimated using linear or polynomials of degree 2 functions with a jacknife resampling method, are for illustration purpose only.

The strongest correlation was observed between food system sustainability and change over time in merchandise and services trade per capita (Fig 2A). This indicator was introduced as a proxy for the internationalization of private investments in food systems. The correlation was found to be positive and strong (*ρ* = 0.70, *p* < 0.0001), suggesting that countries with large increase in merchandise and services trade over the last decade (2004–2015) are also amongst the countries with high sustainability scores. The color-code used to discern between low-, middle- and high-income countries reveals that middle-income countries are amongst those that contribute the most to this strong correlation. Around 3,000 USD of trade per capita the relation starts plateauing and beyond this point (mainly for high-income countries), an increase in trade is not associated with any further increase in food systems’ sustainability.

The second strongest correlation was found with change over time in population growth (Fig 2B). In this case, a strong and negative association with countries’ food system sustainability scores is observed (*ρ* = -0.66, *p* < 0.0001), suggesting that the countries which have experienced the fastest growth in population in the last decade are also amongst those with the lowest food sustainability scores. The figure indicates that those countries are for their majority low-income countries. On the other end of the spectrum, high-income countries, which are characterized by lower levels of population change, are also amongst those displaying higher sustainability scores.

The third correlation is moderate in strength and, again, negative (*ρ* = -0.47, *p* < 0.0001). It concerns the changes over time in agricultural area, used as a proxy for the intensification of agriculture sector (Fig 2C). Countries on the right hand side of the 0 value along the horizontal axis (i.e. with positive values) are countries which have continued to expand their agricultural “frontiers” in the last decade. Those are also amongst the countries with the lowest sustainability scores. The figure indicates that they are mainly middle- and low-income countries. In contrast, countries with negative values (essentially high-income countries) are also characterized by higher sustainability scores.

Two more drivers display Spearman coefficients that are greater than 0.30 and at the same time statistically significant. Those are the change over time in women employment in services (*ρ* = -0.34, *p* = 0.013) and the change over time in urban population (*ρ* = -0.33, *p* = 0.001)–both introduced in the analysis as indicators of urbanization and its associated changes in life style (Fig 2D and 2E). For those two correlations, the relationship is negative, and the distinction low / high income, although still observable, is less marked.

The interpretation of all these different results will be discussed in greater detail in the following section.

Beyond the five drivers mentioned above, four other indicators appear to have statistically significant correlations with the food system sustainability scores. These are: changes in fertilizer use (negative); (ii) change in cereal yield (positive); (iii) change in foreign direct investment (positive), and (iv) change in GDP per capita (negative). Their Spearman coefficients are below 0.30 however, indicating that these correlations are relatively weak–see Table 3 for details.

### Principal component analysis

To strengthen our understanding of the relationship between drivers and indicators, a Principal Component Analysis (PCA) was then implemented, with the main objective to verify that the results obtained through the univariate framework of the Spearman correlation were robust and not distorted by the existence of collinearity between drivers. The results of the PCA (performed using the drivers’ correlation matrix) are summarized numerically in Table 4 (eigenvalues and % variance for the 10 first principal components).

**Table 4:** Results of the principal component analysis^(1)^.

	eigenvalue	% of variance	cumulative % of variance
**PC 1**^**(2)**^	2.98	14.17	14.17
**PC 2**	2.11	10.05	24.23
**PC 3**	2.02	9.61	33.84
**PC 4**	1.51	7.21	41.04
**PC 5**	1.47	7.00	48.04
**PC 6**	1.34	6.38	54.42
**PC 7**	1.16	5.53	59.95
**PC 8**	1.12	5.35	65.30
**PC 9**	1.04	4.97	70.27
**PC 10**	0.88	4.21	74.48

^(1)^ Analysis performed on the correlation matrix of the variables (drivers)

^(2)^ PC =principal component.

Table 4 reveals that the first two principal components (PCs) captures only a relatively modest proportion of the overall variance (24%) and that the % of variance along the 10 first PCs is characterized by low and very similar values decreasing only very slowly across the PCs. This suggests that the variables (drivers) are “scattered” in the variable’s space and essentially uncorrelated. This is confirmed by the cross-correlation matrix computed for the 20 drivers (displayed in diagramme in S4 Fig) which shows very little cross-correlations between the drivers. Overall, this indicates that the risk of multi-collinearity in our analysis is minimal and therefore that the Spearman correlation results are robust.

## Discussion

The objective of this research was to build from existing literature and explore more systematically country-level relationships between the sustainability of food systems and major drivers of change in food systems. The underlying rationale for this research was the recognition that without a clear(er) understanding of what drives food system sustainability (or unsustainability), it is difficult to identify the relevant investments and interventions that are needed to steer food systems toward the “great transformation” that many concerned parties are calling for [76, p.476;2,3,^77^].

The global map of food system sustainability used this study includes 97 countries from low, middle, and high-income regions in proportions that are remarkably close to the overall proportions of low, middle, and high-income countries as observed in the world for the period considered. The study extends the previous work on sustainability indexes made in the literature in several ways (see e.g. [6,32,78,79]). First, several of those previous works [e.g. 32,79] use regional values to fill the gaps of missing country-level information for several indicators. The global map calculation developed by [18] and used here relies only on available national-level datasets, thus avoiding regionally extrapolated values that reduce the correctness of the analysis. Second, the computation of the score is designed to capture the four key dimensions of food systems sustainability: food security & nutrition, environment, social, and economic dimensions [18]. This is an important conceptual improvement since most of the current analyses focus only on one–or at best two–dimensions of sustainability. For instance, the EAT-lancet report [76] considers food security & nutrition and the environment, but fails to incorporate adequately the social and economic elements of food systems in their analysis.

Several conceptual or technical issues limit the conclusions of this work. The first and perhaps most important one relates to the poor data availability that characterizes some components of food systems. This lack of data has some implications for our analysis, in that the sustainability scores as computed here and in [18] capture better the (un)sustainability around the agriculture sector than around the other sectors of the food systems–in particular the sectors of transformation, transport, retail and distribution of food. This issue also means that, at the present time, the number of indicators is not equal across the different dimensions of the metric. While the environmental and food security & nutrition dimensions are represented by a reasonable number of indicators, the diversity of indicators currently available in relation to the economic and social dimensions of food systems is still extremely low. A similar limitation affects the computation of the drivers. The availability of the data used to estimate the drivers in certain countries is poor. As a consequence the dynamics and changes characterizing those components are still poorly captured. This issue highlights the urgent need for strategically positioned stakeholders (donors, international development agencies, etc.) to work more actively with governments toward a more comprehensive monitoring of food systems activities, especially in those domains where data are still missing.

Despite those limitations, this study offers some important contribution to the current research on food system. First, it relies on the first map that explicitly and rigorously incorporates all four dimensions of food systems’ sustainability and that offers a full, systemic assessment of the sustainability of our food systems at a global scale. This map, presented in [18], is used here for the first time in a global analysis.

The second major contribution of this work is the quantitative nature of the analysis around drivers of food systems. While several previous studies highlighted the importance of food system drivers (see e.g., [3,5,27,28,74,80]), none of them attempted to actually quantify the impacts of those drivers on food systems and/or on the sustainability of food systems. The identification of 12 key drivers and their associated indicators allowed us to explore more rigorously this question.

The analysis shows that very few of these drivers display both significant and positive associations with food system sustainability. Except for changes in trade flows of merchandise and services per capita (Fig 2A), changes in population growth, agricultural area, female employment in services, and urban population are all negatively associated with country food system sustainability scores; any further increase in those drivers is expected to be associated with a further decrease in the overall sustainability of our food systems.

Change in trade–effectively a proxy for internationalization of private investments–appears to be positively associated with food system sustainability. While weak, a positive association was also found with the indicators of changes in foreign direct investment and changes in food exports (cf. Table 3). Increasing trade can signify many things, including “globalization”, structural transformation, economic development related to the roles of agriculture, manufacturing and services, and openness to external and sometimes, international markets [81]. Trade can have positive impacts on food systems including improved international commodity prices, higher agricultural output growth, increased food diversity, and stronger knowledge exchange [82–84]. These food system-related economic activities bring benefits such as employment, food security, and further investment opportunities. Trade can, however, also have negative consequences on some dimensions of sustainability such as facilitating unhealthy dietary patterns and non-communicable diseases associated with nutrition transition [85–89] as well as increasing pressure on natural resources and subsequent environmental degradation [90]. In our case, while the relation appears positive overall and significantly stronger for middle-income countries, it weakens substantially for the higher values of trade flows observed for higher-income countries, suggesting that there may be an upper limit to the sustainability benefits offered by trade.

Our analysis also shows that countries with fast growing populations struggle to keep their food systems sustainable (Fig 2B). Increasing population puts pressure on food systems, with a larger number of people to feed exacerbated by a number of other factors, including the increase in consumption per capita, a changing diet associated with lifestyle transition, urbanization and demographic dynamics, and aging populations in places such as Asia and Europe, or youth bulges in Africa [27,91]. While some of those trends have been highlighted in the past, e.g. [85,92], it was generally with a focus on one domain (e.g. health/nutrition or environment–[76,93]). Very little empirical evidence has been offered that builds on, and embraces simultaneously, the four dimensions of the system. The global scale that characterizes this analysis also allowed us to explore those questions across the whole economic development spectrum, from low-income to high-income countries. This revealed the often sensitive situation of those low- and middle-income countries which generally appear to be associated with the lower values of the sustainability scores.

In this global and holistic analysis, another driver that deserves greater in-depth examination is the transition in lifestyle as measured by the change in female employment in services (Fig 2D). Our analysis indicates that women’s transition away from domestic duties to formal work (outside home) is negatively associated with food system sustainability and this effect seems to affect everyone: low, middle but also high-income countries. This trend is likely to be due to changes in both women’s time and work burdens in caring for children and consumption patterns such as reducing available time for meal shopping and cooking, increasing demand for time-saving food preparation, rising intake of meals consumed away from home, and more pre-packaged and easy to consume convenience foods which become now available in a growing number of countries–high, middle but also lower-income countries [23,93].

In spite of the potential economies of scale associated with the concentration of populations occurring in urban areas, urbanization itself also appears in this study to be negatively associated with food system sustainability (Fig 2E). In many parts of the world, there is an expansion of cities into peri-urban and rural communities as well as “ruralized” urban areas and “urbanized” rural landscapes [94,95]. There is, and will continue to be, a loss of agricultural land due to urban expansion: 1.8–2.4% loss of global croplands by 2030, with substantial regional disparities– 80% of global cropland loss from urban expansion in Asia and Africa [96]. Urbanization however also brings changes towards more employment in the goods and services sectors [94], which we argue has positive impacts up to a degree. Urban populations have access to higher food environment diversity but also to more animal source foods (i.e. meat and dairy) and more (ultra)processed and packaged convenient food products [97,98]. These foods require higher income and can be both healthy and less healthy [2,92,99,100], thus creating several different pressures from the same driver. Diets of urban and higher-income societies have also often a larger environmental footprint and are therefore likely to entail more natural resources [101,102]. Additional exploration is needed to understand what latent factors influence positive and negative outcomes associated with this key driver.

Perhaps unsurprisingly, countries that continue to expand their agricultural frontier are associated with unsustainable food systems (Fig 2C). With changes in the types of foods consumers demand, land expansion paired with deforestation is likely to continue [43,75,103]. Among the key drivers discussed so far, this driver is probably the one with the most direct relationship with the supply side of the system, and as such it may have direct implications for the environmental dimensions of the food system sustainability index. Our analysis highlights (once again) the North/South disparity of this dynamics, where land and forest continue to be converted (grabbed?) essentially in the South.

In addition to those key drivers for which the analysis reveals noticeable and statistically robust correlations with the level of sustainability of countries’ food systems, the analysis also uncovers some more unexpected insights into the complex, interconnected nature of food systems. In particular a series of other drivers which are generally recognized in the literature as important sources of food system changes did not show any statistically significant correlation with the sustainability scores. Amongst those are the indicators on climate change (Table 3). Despite the fact that a large body of evidence demonstrates that the impact of climate change on agriculture is already substantial and expected to be even more severe in the future [59,104,105], none of the four related indicators included in our analysis showed significant association with the level of food system (un)sustainability. The most likely explanation for this is that the well-established impact of climate-related extreme events and increases in temperature and rainfall on the productivity of crops in certain regions of the world has not yet translated into net effects on the structure or functioning of the wider food systems which, over the period analyzed here, has managed to "absorb" those disturbances. The future capacity of the global food system to continue to absorb major shocks in regional and local food systems is uncertain, however. A similar type of explanation may apply to a second driver that is also rapidly emerging, which is the raising attention paid to diet and health. Although this increasing trend is central to the “great transformation” of food systems towards sustainability [3,9,106], up to now, only marginal changes have been observed, essentially in niche-markets in high-income countries [45–47]. Those changes do not seem, however, to be “transformative” enough to overturn the current trends, even if it is now recognized that diets can change faster than we originally thought [107].

Overall, four out of the five main drivers of food system sustainability (namely, increase in population growth, increase in agricultural land use, increase in female employment and increase in urbanization) have negative associations with the sustainability of the food system. With, perhaps, the exception of trends associated with agricultural land expansion, all these drivers are characterized by trends with strong inertia, i.e. dynamics that will be difficult to curb down or to contain, and possibly with positive feedback effects (dynamics that amplify and build on their own effect as they evolves). For instance, there is no quick and easy solution to curtail population growth even with substantial and continued investments across a wide range of sectors and actors. Similarly, urbanization and the associated changes in life style—including the increased involvement of women in economic activities–are structural transitions that would be difficult to alter or to stop. The only strong positive driver is the change in trade exchange, but the analysis reveals that this influence rapidly plateaus, meaning that one cannot hope to “push” it too far to compensate for other negative drivers. The analysis already shows that as far as high-income countries are concerned the potential effect is already attenuated. Combined together, these trends suggest therefore that, over the course of the next decade(s), these negative relationships are likely to continue–at least at the global level. This might be further exacerbated by other emerging dynamics given that, at present, the negative effects of climate change on food systems have not yet been fully felt. In this context, one of the most direct countermeasure that is garnering attention is the consumer shift toward more sustainable diets [107]; but the attempts to cultivate sustainable diet-related behavioral changes are still in their infancy and their impacts yet to be realized.

## Conclusion

The initial motivation for this work was the recognition that without a better understanding of what drives the sustainability of our food systems it will be difficult to provide decision-makers with policy-relevant information. Our ambition was therefore to explore more thoroughly the interactions between the sustainability of food systems and the key drivers that are shaping those systems at a global scale.

The analysis highlights how the majority of drivers that show moderate or strong associations with food system sustainability are at present displaying negative correlations. This finding substantiates the consensus currently emerging amongst both national and international expert communities about the urgent need to ‘‘change the current trajectory of the food systems” [108, p. 24]. The analysis also reveals the magnitude of the challenge, with the drivers presently contributing the most to the negative trends being directly related the global-scale demographic transition of the world population (urbanization, population growth, changes in life-style). These drivers are external to food systems, have taken many years to consolidate, may be lagged in effect, and are very slow to respond to “levers” of change.

While this analysis is one of the first efforts to provide quantitative evidence at a global scale of significant correlations between the unsustainability of our food systems and particular drivers, it represents only an initial step toward a more comprehensive and more dynamic understanding of the present and future transformations of these systems. More research is needed to identify a concrete policy and action road map that can help reversing those negative trends.

## Supporting information

S1 Table List of 83 documents identified through the initial screening step. (DOCX)

S2 Table Inclusion / exclusion criteria used for the selection of sustainability indicators. (DOCX)

S3 Table The 27 indicators used as proxies for the sustainability of food systems, and their definitions. (DOCX)

S4 Table Sustainability score for the 97 countries based on 20 indicators. (DOCX)

S5 Table Detailed definitions of all drivers‘ indicators as listed in Table 2. (DOCX)

S1 Fig PRISMA flow diagram describing the selection process regarding the indicators to be included in the computation of the final food system sustainability score. (DOCX)

S2 Fig Spearman-correlation matrix of the 27 indicators included in the food system sustainability metric. High positive correlations are indicated in dark blue, while high negative correlations are showed in dark red. The diagram shows that the environmental and social dimensions are characterized by low internal cross-correlations, while the economic and food & nutrition dimensions display a larger number of high positive and/or negative cross-correlations.
(DOCX)

S3 Fig Trade-off ‘frontier’ between the number of countries for which the indicator datasets are complete and the number of indicators included in the metric. The frontier shows that the larger the number of indicators considered, the smaller the number of countries for which those indicator datasets are complete, and vice versa.
(DOCX)

S4 Fig Cross-correlation matrix of the 20 drivers’ indicators. High positive correlations are indicated in dark blue, while high negative correlations are showed in dark red. The diagram shows that very little cross-correlations occur.
(DOCX)
