# Supplementary material for: Global drivers of food system (un)sustainability: A multi-country correlation analysis
Source: PLoS One. 2020 Apr 3;15(4):e0231071. doi: 10.1371/journal.pone.0231071 (PMC7122815; doi:10.1371/journal.pone.0231071)
Supplement: S1 Table — (DOCX) [file pone.0231071.s001.docx]

S1 Table. List of 83 documents identified through the initial screening step.

|  | References |
| --- | --- |
| 1 | Allen T. & P. Prosperi. (2014). Metrics of Sustainable Diets and Food Systems. Workshop Report. Bioversity International & CIHEAM-IAMM. Montpellier, France. Available online at: www.bioversityinternational.org |
| 2 | Allen, T., & Prosperi, P. (2016). Modeling Sustainable Food Systems. Environmental Management, 57(5), 956-975. https://doi.org/10.1007/s00267-016-0664-8 |
| 3 | Bella, D.A., Jacobs, R. & Li, H. Environmental Management (1994) 18: 489. https://doi-org.ezproxy.unal.edu.co/10.1007/BF02400854 |
| 4 | BerdeguéJ. A. andProctor F. J.withCazzuffi C., (2014). Inclusive Rural–Urban Linkages. Working Paper Series N° 123. Working Group: Development with Territorial Cohesion.Territorial Cohesion for Development Program. Rimisp, Santiago, Chile. https://www.researchgate.net/publication/270899633_Inclusive_Rural-Urban_Linkages |
| 5 | Bernstein, H. (2016). Agrarian political economy and modern world capitalism: the contributions of food regime analysis. The Journal of Peasant Studies, 43(3), 611-647. https://doi.org/10.1080/03066150.2015.1101456 |
| 6 | Biel, R. Sustainable Food Systems. (2016). London, UCL Press. http://dx.doi.org/10.14324/111.9781911307099 |
| 7 | Borelli S. and Lenzerini F. (ed.). (2012). Cultural Heritage, cultural rights, cultural diversity : new developments in international law. Martinus Nijhoff Publishers. Leiden, Boston. |
| 8 | Borras, S. M., Franco, J. C., Isakson, S. R., Levidow, L., & Vervest, P. (2016). The rise of flex crops and commodities: implications for research. Journal of Peasant Studies, 43, 93-115. https://doi.org/10.1080/03066150.2015.1036417 |
| 9 | Brunner, T. A., van der Horst, K., & Siegrist, M. (2010). Convenience food products. Drivers for consumption. Appetite, 55(3), 498-506. https://doi.org/10.1016/j.appet.2010.08.017 |
| 10 | Cafiero C., Melgar-Quiñonez H.R., Ballard T.J., Kepple A.W. (2014). Validity and reliability of food security measures. Annals of the New York Academy of Sciences. Dec 1;1331(1):230-48 |
| 11 | CGIAR Consortium Office, EAT Initiative, Sustainable Development Solutions Network. 2015. Integrated Indicators for Sustainable Food Systems and Healthy Diets in the Post-2015 Development Agenda_17 Sept Final Statement. https://hdl.handle.net/10947/401 |
| 12 | Drewnowski, A., Rehm, C. D., Martin, A., Verger, E. O., Voinnesson, M., & Imbert, P. (2015). Energy and nutrient density of foods in relation to their carbon footprint. The American Journal of Clinical Nutrition, 101(1), 184-191. https://doi.org/10.3945/ajcn.114.092486 |
| 13 | Dubé, L., Webb, P. , Arora, N. K. and Pingali, P. (2014), Agriculture, health, and wealth convergence: bridging traditional food systems and modern agribusiness solutions. Ann. N.Y. Acad. Sci., 1331: 1-14. doi:10.1111/nyas.12602 |
| 14 | Eakin, H., Connors, J.P., Wharton, C., Bertmann, F., Xiong, A., Stoltzfus, . (2017). Identifying attributes of food system sustainability: emergingthemes and consensu. Agric Hum Values, 34:757–773. DOI 10.1007/s10460-016-9754-8 |
| 15 | Economist Intelligence Unit (EIU) and Barilla Center for Food and Nutrition (BCFN). (2016). Fixing food: towards a more sustainable food system. http://foodsustainability.eiu.com/whitepaper/ |
| 16 | Ehrich, M., & Mangelsdorf, A. (2018). The Role of Private Standards for Manufactured Food Exports from Developing Countries. World Development, 101, 16-27. https://doi.org/10.1016/j.worlddev.2017.08.004 |
| 17 | Ericksen, P. J. (2008). Conceptualizing food systems for global environmental change research. Global Environmental Change, 18(1), 234-245. https://doi.org/10.1016/j.gloenvcha.2007.09.002 |
| 18 | Ericksen, P., Bohle, H.-G., Stewart, B., (2010). Vulnerability and resilience of foodsystems. In: Ingram, I., Erickson, P., Liverman, D. (Eds.), Food Security and Global Environmental Change. Earthscan, London. |
| 19 | Fanzo, J., Cogill, B., and F. Mattei (2012). Metrics of Sustainable Diets and Food Systems. Bioversity International, Rome, Italy. https://www.bioversityinternational.org/fileadmin/_migrated/uploads/tx_news/Metrics_of_sustainable_diets_and_food_systems_1572.pdf |
| 20 | FAO 2015. Climate change and food systems: global assessments and implications for food security and trade. Food Agriculture Organization of the United Nations (FAO). http://www.fao.org/3/a-i4332e.pdf |
| 21 | FAO, IFAD and WFP. (2015). The State of Food Insecurity in the World 2015. Meeting the 2015 international hunger targets: taking stock of uneven progress.Rome, FAO |
| 22 | FAO, IFAD, UNICEF, WFP and WHO. 2018. The State of Food Security and Nutrition in the World 2018. Building climate resilience for food security and nutrition. Rome, FAO. http://www.fao.org/3/i9553en/i9553en.pdf |
| 23 | FAO.2014. Developing sustainable food value chains – Guiding principles. Rome. www.fao.org/3/a-i3953e.pdf |
| 24 | FAO.2014. SAFA, Sustainability Assessment of Food and Agriculture Systems. Version 3.0. Rome. http://www.fao.org/3/a-i3957e.pdf |
| 25 | FAO.2016. Influencing food environments for healthy diets. Rome. www.fao.org/3/a-i6484e.pdf |
| 26 | Fresco L, Poppe K (2016) Towards a common agricultural and food policy. Wageningen University and Research,Wageningen. doi:10.18174/390280. |
| 27 | Friedmann, H. (2015). Governing land and landscapes: Political ecology of enclosures and commons. Canadian Food Studies / La Revue Canadienne Des Études Sur l’alimentation, 2(2), 23-31. https://doi.org/10.15353/cfs-rcea.v2i2.95 |
| 28 | Frison, E. A., Cherfas, J., & Hodgkin, T. (2011). Agricultural Biodiversity Is Essential for a Sustainable Improvement in Food and Nutrition Security. Sustainability, 3(1), 238-253. https://doi.org/10.3390/su3010238 |
| 29 | Frison, E.A.; IPES-Food. (2016) From uniformity to diversity: a paradigm shift from industrial agriculture to diversified agroecological systems. Louvain-la-Neuve (Belgium): IPES, 96 p. https://hdl.handle.net/10568/75659 |
| 30 | Global Panel on Agriculture and Food Systems for Nutrition. 2016. Food systems and diets: Facing the challenges of the 21st century. London, UK. |
| 31 | Godfray H. Charles J., Crute Ian R., Haddad Lawrence, Lawrence David, Muir James F., Nisbett Nicholas, … Whiteley Rosalind. (2010). The future of the global food system. Philosophical Transactions of the Royal Society B: Biological Sciences, 365(1554), 2769-2777. https://doi.org/10.1098/rstb.2010.0180 |
| 32 | Gómez, M. I., & Ricketts, K. D. (2013). Food value chain transformations in developing countries: Selected hypotheses on nutritional implications. Food Policy, 42, 139-150. https://doi.org/10.1016/j.foodpol.2013.06.010 |
| 33 | Grando, S., Rahmanian, M., Colombo, L. (2016). TRANSMANGO. Matrix on the multidimensionality of vulnerabilities and their drivers |
| 34 | Gussow, J. D., & Clancy, K. L. (1986). Dietary guidelines for sustainability. Journal of Nutrition Education (USA). Retrieved from http://agris.fao.org/agris-search/search.do?recordID=US881472388 |
| 35 | Gustafson, D., Gutman, A., Leet, W., Drewnowski, A., Fanzo, J., & Ingram, J. (2016). Seven Food System Metrics of Sustainable Nutrition Security. Sustainability, 8(3), 196. https://doi.org/10.3390/su8030196 |
| 36 | Guyomard, H., Darcy-Vrillon, B., Esnouf, C., Marin, M., Russel, M., & Guillou, M. (2012). Eating patterns and food systems: critical knowledge requirements for policy design and implementation. Agriculture & Food Security, 1(1), 13. https://doi.org/10.1186/2048-7010-1-13 |
| 37 | Haddad, L., Hawkes, C. ORCID: 0000-0002-5091-878X, Waage, J., Webb, P., Godfray, C. and Toulmin, C. (2016). Food systems and diets: Facing the challenges of the 21st century. London, UK: Global Panel on Agriculture and Food Systems for Nutrition. http://openaccess.city.ac.uk/19323/ |
| 38 | Hammelman, C., & Hayes-Conroy, A. (2015). Understanding Cultural Acceptability for Urban Food Policy. Journal of Planning Literature, 30(1), 37–48. https://doi.org/10.1177/0885412214555433 |
| 39 | Hawkes, C., Friel, S., Lobstein, T., & Lang, T. (2012). Linking agricultural policies with obesity and noncommunicable diseases: A new perspective for a globalising world. Food Policy, 37(3), 343–353. https://doi.org/10.1016/j.foodpol.2012.02.011 |
| 40 | Herforth, A., & Ahmed, S. (2015). The food environment, its effects on dietary consumption, and potential for measurement within agriculture-nutrition interventions. Food Security, 7, 505-520. https://doi.org/10.1007/s12571-015-0455-8 |
| 41 | HLPE. 2017. Nutrition and food systems. A report by the High Level Panel of Experts on Food Security and Nutrition of the Committee on World Food Security, Rome |
| 42 | ILSI Research Foundation Scientific Session. (2017). Hungry Cities: The global revolution in food systems. ILSI Annual Meeting 2017. January 23, 2017. Hilton La Jolla Torrey Pines, California. Retrieved from: https://ilsirf.org/event/hungry-cities-the-global-revolution-in-food-systems/ |
| 43 | Ingram, J. (2011). A food systems approach to researching food security and its interactions with global environmental change. Food Sec. 3: 417. https://doi-org.ezproxy.unal.edu.co/10.1007/s12571-011-0149-9 |
| 44 | Institute of Medicine. (2012). Improving Food Safety Through a One Health Approach: Workshop Summary. Washington, DC: The National Academies Press. https://doi.org/10.17226/13423. |
| 45 | International Food Policy Research Institute (IFPRI). 2016. Global Food Policy Report. Washington, D.C.: International Food Policy Research Institute (IFPRI). http://dx.doi.org/10.2499/9780896295827 |
| 46 | IOM (Institute of Medicine) and NRC (National Research Council). (2015). A framework for assessing effects of the food system. Washington, DC: The National Academies Press. |
| 47 | Johnston, J. L., Fanzo, J. C., & Cogill, B. (2014). Understanding sustainable diets: a descriptive analysis of the determinants and processes that influence diets and their impact on health, food security, and environmental sustainability. Advances in nutrition (Bethesda, Md.), 5(4), 418–429. doi:10.3945/an.113.005553 |
| 48 | Jones, A. D., Hoey, L., Blesh, J., Miller, L., Green, A., Fink Shapiro, L.,. (2016). A Systematic Review of the Measurement of Sustainable Diets, Advances in Nutrition, Volume 7, Issue 4, July 2016, Pages 641–664, https://doi.org/10.3945/an.115.011015 |
| 49 | Jones, A. D., Shrinivas, A., & Bezner-Kerr, R. (2014). Farm production diversity is associated with greater household dietary diversity in Malawi: Findings from nationally representative data. Food Policy, 46, 1-12. https://doi.org/10.1016/j.foodpol.2014.02.001 |
| 50 | Kittler, P. G., Sucher, K. P., Nahikian-Nelms, M. (2012). Food and Culture. Sixth edition. Wadsworth, Cengage Learning. Retrieved from http://people.wku.edu/barry.kaufkins/330/Food%20and%20Culture.pdf |
| 51 | Macdiarmid, J. I. (2013). Is a healthy diet an environmentally sustainable diet? The Proceedings of the Nutrition Society, 72(1), 13-20. https://doi.org/10.1017/S0029665112002893 |
| 52 | Macfadyen, S., Tylianakis, J. M., Letourneau, D. K., Benton, T. G., Tittonell, P., Perring, M. P., … Smith, H. G. (2015). The role of food retailers in improving resilience in global food supply. Global Food Security, 7, 1-8. https://doi.org/10.1016/j.gfs.2016.01.001 |
| 53 | Masters, W.A., A. Hall, E.M. Martinez, P. Shi, G. Singh, P. Webb and D. Mozaffarian, 2016. The nutrition transition and agricultural transformation: a Preston curve approach. Agricultural Economics, 47(S1), pp. 97-114. https://doi.org/10.1111/agec.12303 |
| 54 | McMichael, P. (2009). A food regime genealogy. The Journal of Peasant Studies, 36(1), 139-169. https://doi.org/10.1080/03066150902820354 |
| 55 | Meagan E. Schipanski, Graham K. MacDonald, Steven Rosenzweig, M. Jahi Chappell, Elena M. Bennett, Rachel Bezner Kerr, Jennifer Blesh, Timothy Crews, Laurie Drinkwater, Jonathan G. Lundgren, Cassandra Schnarr. Realizing Resilient Food Systems. BioScience, Volume 66, Issue 7, 01 July 2016, Pages 600–610, https://doi.org/10.1093/biosci/biw052 |
| 56 | Melesse, M. B., Van Den Berg, M. (2017). Food Systems Metrics and Methods: A Synthesis. Development Economics Group, Wageningen University, Wageningen, the Netherlands |
| 57 | Ndamani, F., & Watanabe, T. (2017). Developing indicators for adaptation decision-making under climate change in agriculture: A proposed evaluation model. Ecological indicators, 76, 366-375. doi: 10.1016/j.ecolind.2016.12.012 |
| 58 | Nea, F. M., Kearney, J., Livingstone, M. B. E., Pourshahidi, L. K., & Corish, C. A. (2015). Dietary and lifestyle habits and the associated health risks in shift workers. Nutrition Research Reviews, 28(2), 143–166. https://doi.org/10.1017/S095442241500013X |
| 59 | Notarnicola, B., Sala, S., Anton, A., McLaren, S. J., Saouter, E., & Sonesson, U. (2017). The role of life cycle assessment in supporting sustainable agri-food systems: A review of the challenges. Journal of Cleaner Production, 140, 399–409. https://doi.org/10.1016/j.jclepro.2016.06.071 |
| 60 | Nyerges, T., Roderick, M., Prager, S., Bennett, D., & Lam, N. (2014). Foundations of sustainability information representation theory: spatial–temporal dynamics of sustainable systems. International Journal of Geographical Information Science, 28(5), 1165-1185. https://doi.org/10.1080/13658816.2013.853304 |
| 61 | Padilla M, Cogill B, Prosperi P, Allen T, Peri I (2015) Metrics for sustainable diets and food systems: insights from a multi-institutional research partnership. In: Innovative indicators and initiatives for Food Security: Feeding Expo Milano with Mediterranean perspectives. CIHEAM Watch Letters N.32, Paris. www.ciheam.org/publications/191/011_-_Prosperi.pdf |
| 62 | Pingali, P.L. & K.D. Ricketts. 2014. Mainstreaming nutrition metrics in household surveys—toward a multidisciplinary convergence of data systems. Ann. N.Y. Acad. Sci.1331:249–257. doi: 10.1111/nyas.12597 |
| 63 | Prescott, J., & Bell, G. (1995). Cross-Cultural Determinants of Food Acceptability - Recent Research on Sensory Perceptions and Preferences. Trends in Food Science & Technology, 6, 201–205. https://doi.org/10.1016/S0924-2244(00)89055-X |
| 64 | Prosperi, P., Allen, T., Cogill, B. T., Padilla, M., Peri, L. (2016) Towards metrics of sustainable food systems: a review of the resilience and vulnerability literature. Environ Syst Decis (2016) 36: 3. https://doi-org.ezproxy.unal.edu.co/10.1007/s10669-016-9584-7 |
| 65 | Prosperi, P., Allen, T., Padilla, M., Peri, L., & Cogill, B. (2014). Sustainability and Food & Nutrition Security: A Vulnerability Assessment Framework for the Mediterranean Region. SAGE Open, 4(2), 2158244014539169. https://doi.org/10.1177/2158244014539169 |
| 66 | Ranganathan, J. et al. 2016. Shifting Diets for a Sustainable Food Future.” Working Paper, Installment 11 of Creating a Sustainable Food Future. Washington, DC: World Resources Institute. Accessible at http://www.worldresourcesreport.org. |
| 67 | Rawlinson, M. and C., Ward, C., (eds.). 2017. The Routledge Handbook of Food Ethics. Routledge Taylor and Francis Group. New York. |
| 68 | Reardon, T., & Timmer, C. P. (2012). The Economics of the Food System Revolution. Annual Review of Resource Economics, 4(1), 225-264. https://doi.org/10.1146/annurev.resource.050708.144147 |
| 69 | Roever, S., & Skinner, C. (2016). Street vendors and cities. Environment and Urbanization, 28(2), 359-374. https://doi.org/10.1177/0956247816653898 |
| 70 | Sampson, D., and Wills, C. 2013. Culturally appropriate food: Researching cultural aspects of food sovereignty. Conference paper for discussion at: Food Sovereignty: A Critical Dialogue International Conference. Yale University, September 14-15, 2013. Retrieved from: https://www.tni.org/en/briefing/culturally-appropriate-food |
| 71 | Schwarz, J., Schuster, M., Annaert, B., Maertens, M., & Mathijs, E. (2016). Sustainability of global and local food value chains. An empirical comparison of Peruvian and Belgian asparagus (Working Papers N.o 232594). Retrieved from Katholieke Universiteit Leuven, Centre for Agricultural and Food Economics website: https://EconPapers.repec.org/RePEc:ags:kucawp:232594 |
| 72 | The Food Foundation. (2016). Food Environment Policy Index (Food-Epi) for England. Final version. November 20016. https://foodfoundation.org.uk/wp-content/uploads/2016/11/ENGLAND-Food-EPI-Report-FINAL1.pdf |
| 73 | Thompson, J., Millstone, E., Scoones, I., Ely, A., Marshall, F., Shah, E. and Stagl, S. (2007) Agri-food System Dynamics: pathways to sustainability in an era of uncertainty, STEPS Working Paper 4, Brighton: STEPS Centre. https://www.ids.ac.uk/files/agriculture.pdf |
| 74 | Tilman, D., & Clark, M. (2014). Global diets link environmental sustainability and human health. Nature, 515, 518. doi:10.1038/nature13959 |
| 75 | UN Human Rights Council, Report of the Special Rapporteur on the Right to Food, Olivier de Schutter. 26 December 2011, A/HRC/19/59 https://europa.eu/capacity4dev/file/13085/download?token=Y-XFyCJo |
| 76 | UNEP (2016) Food Systems and Natural Resources. A Report of the Working Group on Food Systems of the International Resource Panel. Westhoek, H, Ingram J., Van Berkum, S., Özay, L., and Hajer M.Job Number: DTI/1982/PAISBN: 978-92-807-3560-4 |
| 77 | UNESCO. Towards a UNESCO culture and development indicators suite. 2005. Working document. Dimensionn°5:Culturaldimensioninhumanrightsanddevelopment. |
| 78 | Vandevijvere, S., & Swinburn, B. (2014). Creating healthy food environments through global benchmarking of government nutrition policies and food industry practices. Archives of public health, 72(1), 7. doi:10.1186/2049-3258-72-7 |
| 79 | Wise, T.A., 2015. Two roads diverged in the food cri-sis: Global policy takes the one more travelled. Canadian Food Studies / La Revue canadienne des études sur l’alimentation Vol. 2, No. 2, pp. 9–16. https://doi.org/10.15353/cfs-rcea.v2i2.98 |
| 80 | Woodley, E., Crowley, E., Dookie, C., y Carmen, A. (2006). Cultural indicators of Indigenous Peoples' food and agro-ecological systems. Food and Agriculture Organization (FAO) and International Indian Treaty Council (IITC), Puerto Cabezas, Nicaragua: Second Global Consultation on the Right to Food and Food Sovereignty for Indigenous Peoples. Retrieved from: http://www.fao.org/fileadmin/templates/esw/esw_new/documents/SARD/Publications/7_CulturalIndicators_exsum16oct.pdf |
| 81 | World Bank. 2016. World development indicators 2016 (English). World Development Indicators. Washington, D.C. : World Bank Group. http://documents.worldbank.org/curated/en/805371467990952829/World-development-indicators-2016 |
| 82 | World Economic Forum. (2017). Shaping the Future of Global Food Systems: A Scenarios Analysis. http://www3.weforum.org/docs/IP/2016/NVA/WEF_FSA_FutureofGlobalFoodSystems.pdf |
| 83 | WWF. 2016. Living Planet Report 2016. Risk and resilience in a new era. WWW International, Gland, Switzerland |
