# Supplementary material for: Global drivers of food system (un)sustainability: A multi-country correlation analysis
Source: PLoS One. 2020 Apr 3;15(4):e0231071. doi: 10.1371/journal.pone.0231071 (PMC7122815; doi:10.1371/journal.pone.0231071)
Supplement: S2 Table — (DOCX) [file pone.0231071.s002.docx]

S2 Table. Inclusion / exclusion criteria used for the selection of sustainability indicators

- **Cross correlation**. Were *excluded* indicators which are closely cross-correlated to another indicator already considered in the list. For instance, “proportion of population under global poverty line” and “percentage of population living under the poverty threshold” are very closely correlated. We would only keep one of those two indicators.
- **Conceptual relevance**. Were *included* indicators that could clearly be related to one of the four dimensions of the metric, that is: ecological, economic, social and food and nutrition dimensions – see also composite indicator criterion below.
- **Global scale**. Were *included* only indicators for which a database which covers at least 70 countries is available.
- **Global validity**. Were *excluded* indicators that refer to processes that are specific to some specific regions of the world. For instance, “Percentage of agricultural land lost yearly to desertification” was excluded as desertification is a phenomenon that by definition can only occur in some specific regions of the world.
- **Time period**. Were *excluded* indicators for which the database had information only prior to the year 2000.
- **Latent variables**. Were *excluded* indicators that are based on latent variables. For instance, indicators of “resilience” or “economic vulnerability” were excluded as there is no agreed measure/unit of resilience or economic vulnerability.
- **Clear methodology**. Were *excluded* indicators for which the methodology used to construct the database was not clearly detailed in the original database.
- **Non-composite indicators**. Were *excluded* indicators based on composite indices that fall into two different dimensions of the metric. For instance, the ratio “natural capital used / GDP” which is sometimes proposed in the literature as an indicator of sustainability would not be included as it clearly lies at the interface between the environmental and economic dimensions.
- **Comparability**. Were *excluded* (or amended) indicators that were based on absolute numbers that do not allow for comparison between countries – for instance the total number of km of paved roads would not be included. Instead the road density was considered, that is, the total number of km of paved road per 100 square km of land area.
