# Supplementary material for: Global drivers of food system (un)sustainability: A multi-country correlation analysis
Source: PLoS One. 2020 Apr 3;15(4):e0231071. doi: 10.1371/journal.pone.0231071 (PMC7122815; doi:10.1371/journal.pone.0231071)
Supplement: S3 Table — (DOCX) [file pone.0231071.s003.docx]

S3 Table. The 27 indicators used as proxies for the sustainability of food systems, and their definitions

| Indicators | Definition |
| --- | --- |
| Greenhouse gas emissions in total agriculture (gigagrams) | The Emissions by sector domain of the FAOSTAT Agri-Environmental Indicators section contains data on emissions of greenhouse gases (GHG) by gas, economic sector, country and year. It also displays the shares of each sector in the total emissions of each gas (e.g. share of agriculture in total CH4 emissions) and the shares of each gas in the emissions from each sector (e.g. share of CH4 in the emissions from Agriculture). |
| Water pH (pH units) | The negative logarithm of the hydrogen ion concentration, a measure of acidity or alkalinity of water soluble substances. pH values were transformed applying the following formula: \|pH - 7\|, taking 7 as a point reference of good water quality conditions. The values which are above or below this reference point are considered to decline water quality. |
| Agricultural water withdrawal as percentage of total renewable water (%) | Agricultural water withdrawal as percentage of total water withdrawal. [Agricultural water withdrawal as % of total water withdrawal] = [Agricultural water withdrawal]/[Total water withdrawal]*100 |
| Soil carbon (Average carbon content in the topsoil as percentage in weight) | Average carbon content in the topsoil as a % in weight |
| Agricultural land as percentage of arable land (%) | Agricultural area, this category is the sum of areas under “Arable land”, “Permanent crops” and “Permanent pastures”. Arable land is the land under temporary agricultural crops (multiple-cropped areas are counted only once), temporary meadows for mowing or pasture, land under market and kitchen gardens and land temporarily fallow (less than five years). The abandoned land resulting from shifting cultivation is not included in this category. Data for “Arable land” are not meant to indicate the amount of land that is potentially cultivable. |
| Benefits of biodiversity index (0 = no biodiversity potential to 100 = maximum) | The Global Environment Facility’s (GEF) benefits index for biodiversity is a comprehensive indicator of national biodiversity status and is used to guide its biodiversity priorities. For each country the biodiversity indicator incorporates the best available and comparable information in four relevant dimensions: represented species, threatened species, represented ecoregions, and threatened ecoregions. |
| Crop diversity (Calories diversity measured by Shannon index) | Calories diversity measured by Shannon index |
| Agriculture and forestry energy use as percentage of total (%) | Agriculture and forestry energy used as a % of total energy use, measured as Agr.Energy use/Total Energy Use)*100. |
| Agriculture value-added per worker (constant 2010 US$) | Agriculture value added per worker is a measure of agricultural productivity. Value added in agriculture measures the output of the agricultural sector (ISIC divisions 1-5) less the value of intermediate inputs. Agriculture comprises value added from forestry, hunting, and fishing as well as cultivation of crops and livestock production. Data are in constant 2010 U.S. dollars. |
| Agriculture under-employment (%) | The time-related underemployment rate conveys the number of persons in time-related underemployment as a percent of the total number of persons in employment. Persons in time-related underemployment comprise all persons in employment, who satisfy the following three criteria during the reference period: a) are willing to work additional hours; b) are available to work additional hours i.e., are ready, within a specified subsequent period, to work additional hours, given opportunities for additional work; and c) worked less than a threshold relating to working time i.e., persons whose hours actually worked in all jobs during the reference period were below a threshold, to be chosen according to national circumstances. For details, refer to the Resolution concerning the measurement of underemployment and inadequate employment situations. |
| Gini index for land distribution & tendency | The Gini index is a statistical tool that ranges from 0 (indicating perfect equity) to 1 (total inequity). GRAIN gathered Gini indices for agricultural land distribution. |
| Labor force participation rate, female (% of female population ages 15+) (modeled ILO estimate) | Labor force participation rate is the proportion of the population ages 15 and older that is economically active: all people who supply labor for the production of goods and services during a specified period. |
| Predominant fair trade organizations and producers | Predominant number of 1: Fairtrade producer network, 2: Fairtrade organization, 3: Both. Fair trade is an alternative approach to conventional trade based on a partnership between producers and traders, businesses and consumers. The international Fairtrade system - made up of Fairtrade International and its member organizations - represents the world's largest and most recognized fair trade system. |
| Employment in agriculture (% of total employment) | Employment is defined as persons of working age who were engaged in any activity to produce goods or provide services for pay or profit, whether at work during the reference period or not at work due to temporary absence from a job, or to working-time arrangement. The agriculture sector consists of activities in agriculture, hunting, forestry and fishing, in accordance with division 1 (ISIC 2) or categories A-B (ISIC 3) or category A (ISIC 4). |
| Per capita food available for human consumption (kcal/capita/day) | An estimate of the per capita amount of food available for human consumption. |
| Food consumption as share of total income (% of total household expenditure) | A measure of the percentage of household expenditure that is spent on food at a national level. |
| Estimated travel time to the nearest city of 50,000 or more people (Hours travel from a city) | Estimated travel time to the nearest city of 50,000 or more people (Hours travel from a city) |
| Access to improved water resource (%) | It measures the share (%) of the total population with access to improved drinking water sources. Access to an improved water source refers to the percentage of the population with reasonable access to an adequate amount of water from an improved source, such as a household connection, public standpipe, borehole, protected well or spring, and rainwater collection. Unimproved sources include vendors, tanker trucks, and unprotected wells and springs. Reasonable access is defined as the availability of at least 20 liters a person a day from a source within one kilometer of the dwelling. |
| Access to electricity (%) | Access to electricity is the percentage of population with access to electricity. Electrification data are collected from industry, national surveys and international sources. |
| Price volatility index | Monthly variability of the prices (Consumer price index CPI) through a year. |
| Per capita food supply variability (kcal/capita/day) | Per capita food supply variability corresponds to the variability of the "food supply in kcal/caput/day" as disseminated in FAOSTAT. |
| Burden of foodborne illness (number of cases) | Includes estimates of the burden of foodborne diseases caused by 31 bacteria, viruses, parasites, toxins and chemicals. The estimates are based on the best available data at the time of reporting, and identified data gaps were filled using imputation, assumptions and other methods. |
| Food loss as % of total food produced | A measure of post-harvest and pre-consumer food loss as a ratio of the total domestic supply (production, net imports and stock changes) of crops, livestock and fish commodities, in tonnes. |
| Diet diversification | The indicator expresses the low energy supply (in kcal/caput/day) provided by cereals, roots and tubers as a percentage of the total Dietary Energy Supply (DES) (in kcal/caput/day) calculated from the corresponding countries in the FAOSTAT food balance sheets. It is a proxy of diet diversification. |
| Stunting, children aged <5 years stunted (%) | Stunting, children aged <5 years stunted (%) |
| Prevalence of obesity, % of the population, over 18 years of age | Percentage of defined population with a body mass index (BMI) of 30 kg/m2 or higher. |
| Nutrient deficiency, serum retinol deficiency (%) | Serum retinol deficiency (%) |
