# Supplementary material for: Global drivers of food system (un)sustainability: A multi-country correlation analysis
Source: PLoS One. 2020 Apr 3;15(4):e0231071. doi: 10.1371/journal.pone.0231071 (PMC7122815; doi:10.1371/journal.pone.0231071)
Supplement: S4 Table — (DOCX) [file pone.0231071.s004.docx]

S4 Table. Sustainability score for the 97 countries based on 20 indicators

| Countries | Codes | Scores | Countries | Codes | Scores | Countries | Codes | Scores |
| --- | --- | --- | --- | --- | --- | --- | --- | --- |
| New Zealand | NZL | 0.73 | Panama | PAN | 0.57 | Benin | BEN | 0.44 |
| Switzerland | CHE | 0.72 | Romania | ROU | 0.57 | Vietnam | VNM | 0.43 |
| Canada | CAN | 0.72 | Hungary | HUN | 0.56 | Myanmar | MMR | 0.43 |
| United Kingdom | GBR | 0.71 | Chile | CHL | 0.56 | Kenya | KEN | 0.43 |
| Netherlands | NLD | 0.70 | Poland | POL | 0.55 | Laos | LAO | 0.42 |
| United States | USA | 0.70 | Greece | GRC | 0.55 | Zambia | ZMB | 0.41 |
| Austria | AUT | 0.69 | Dominican Republic | DOM | 0.55 | Tanzania | TZA | 0.41 |
| Norway | NOR | 0.69 | Ecuador | ECU | 0.54 | Rwanda | RWA | 0.41 |
| Sweden | SWE | 0.69 | Paraguay | PRY | 0.54 | Cambodia | KHM | 0.39 |
| Australia | AUS | 0.69 | Ukraine | UKR | 0.54 | Tunisia | TUN | 0.38 |
| France | FRA | 0.67 | South Africa | ZAF | 0.53 | Saudi Arabia | SAU | 0.38 |
| Germany | DEU | 0.67 | Peru | PER | 0.53 | Morocco | MAR | 0.38 |
| Spain | ESP | 0.65 | El Salvador | SLV | 0.53 | Burkina Faso | BFA | 0.37 |
| Malaysia | MYS | 0.64 | Mexico | MEX | 0.52 | Nepal | NPL | 0.37 |
| Denmark | DNK | 0.63 | United Arab Emirates | ARE | 0.52 | Mali | MLI | 0.37 |
| Russia | RUS | 0.62 | Nigeria | NGA | 0.52 | Malawi | MWI | 0.37 |
| Finland | FIN | 0.61 | Nicaragua | NIC | 0.51 | Sri Lanka | LKA | 0.36 |
| Kazakhstan | KAZ | 0.60 | Botswana | BWA | 0.51 | Ethiopia | ETH | 0.35 |
| Italy | ITA | 0.60 | Ghana | GHA | 0.51 | Mozambique | MOZ | 0.35 |
| Colombia | COL | 0.60 | Azerbaijan | AZE | 0.51 | Senegal | SEN | 0.32 |
| Japan | JPN | 0.60 | Ivory Coast | CIV | 0.50 | Egypt | EGY | 0.32 |
| Venezuela | VEN | 0.59 | Cameroon | CMR | 0.50 | Niger | NER | 0.32 |
| Bulgaria | BGR | 0.59 | Honduras | HND | 0.50 | Sudan | SDN | 0.31 |
| Slovakia | SVK | 0.59 | Thailand | THA | 0.48 | Algeria | DZA | 0.31 |
| Czechia | CZE | 0.59 | Indonesia | IDN | 0.48 | Yemen | YEM | 0.31 |
| Brazil | BRA | 0.58 | Togo | TGO | 0.47 | Pakistan | PAK | 0.31 |
| South Korea | KOR | 0.58 | Guatemala | GTM | 0.47 | Guinea | GIN | 0.31 |
| Costa Rica | CRI | 0.58 | Philippines | PHL | 0.47 | Madagascar | MDG | 0.30 |
| Uruguay | URY | 0.57 | Turkey | TUR | 0.46 | India | IND | 0.30 |
| Belarus | BLR | 0.57 | Bolivia | BOL | 0.46 | Bangladesh | BGD | 0.29 |
| Kuwait | KWT | 0.57 | Sierra Leone | SLE | 0.46 | Jordan | JOR | 0.26 |
| Belgium | BEL | 0.57 | Tajikistan | TJK | 0.45 |  |  |  |
| Argentina | ARG | 0.57 | Uganda | UGA | 0.44 |  |  |  |
