# Supplementary material for: Global drivers of food system (un)sustainability: A multi-country correlation analysis
Source: PLoS One. 2020 Apr 3;15(4):e0231071. doi: 10.1371/journal.pone.0231071 (PMC7122815; doi:10.1371/journal.pone.0231071)
Supplement: S5 Table — (DOCX) [file pone.0231071.s005.docx]

S5 Table. Detailed definitions of all drivers‘ indicators as listed in Table 2

| **Category** | **Drivers** | **Indicators** | | | **Definition** | | **Database** | | **Period** | | **Selected years** | | **Countries** | | **URL** | |
| --- | --- | --- | --- | --- | --- | --- | --- | --- | --- | --- | --- | --- | --- | --- | --- | --- |
| Demand/ Consumer | Growing attention paid to diet and health | Change over time of interest in aggregate of healthy diet | | | Interest in healthy diet assessed through Google Trend® data between 2004 and 2018 using the aggregated counts (adjusted per capita) for the search of the following key-words : "healthy diet", "junk food", and "organic food" in eight different languages: English, French, Spanish, Portuguese, German, Dutch and Russian. Change over time calculated through the estimation of the linear slope using Sen method. | | Google Trends | | 2004-2018 | | Available data | | 68 | | (1) | |
|  | Population growth | Change over time in annual population growth (%) | | | Annual population growth rate for year t is the exponential rate of growth of midyear population from year t-1 to t, expressed as a percentage. Population is based on the de facto definition of population, which counts all residents regardless of legal status or citizenship. Change over time estimated as the median of the annual population growth rate values calculated between 2004 and 2015. | | World Bank | | 1960-2016 | | 2004-2015 (median) | | 214 | | (2) | |
|  | Raise in consumers' income | Change over time in GDP per capita growth (annual %) | | | Annual percentage growth rate of GDP per capita based on constant local currency. Aggregates are based on constant 2010 U.S. dollars. GDP per capita is gross domestic product divided by midyear population. GDP at purchaser's prices is the sum of gross value added by all resident producers in the economy plus any product taxes and minus any subsidies not included in the value of the products. It is calculated without making deductions for depreciation of fabricated assets or for depletion and degradation of natural resources. Change over time estimated as the median of the annual percentage growth in GDP calculated between 2004 and 2015. | | World Bank | | 2000-2016 | | 2004-2015 (median) | | 192 | | (3) | |
|  | Urbanization | Change over time in urban population (% of total) | | | Urban population refers to people living in urban areas as defined by national statistical offices. The data are collected and smoothed by United Nations Population Division. Change over time estimated as the difference in medians calculated for the periods 2004/2006 and 2014/2016. | | World Bank | | 1960-2016 | | 2004-2016 (2015-2005, median difference) | | 213 | | (4) | |
|  | Women involvement | Change over time in female employment in services (% of female employment) | | | Employment is defined as persons of working age who were engaged in any activity to produce goods or provide services for pay or profit, whether at work during the reference period or not at work due to temporary absence from a job, or to working-time arrangement. The services sector consists of wholesale and retail trade and restaurants and hotels; transport, storage, and communications; financing, insurance, real estate, and business services; and community, social, and personal services, in accordance with divisions 6-9 (ISIC 2) or categories G-Q (ISIC 3) or categories G-U (ISIC 4). Change over time estimated as the difference in medians calculated for the periods 2004/2006 and 2014/2016. | | World Bank | | 1963-2016 | | 2004-2016 (2015-2005, median difference) | | 92 | | (5) | |
| Production/ Supply | Climate change | Change over time in mean temperature (degree Celsius) | | | Mean temperature (degree Celsius). Change over time calculated through linear slope estimation on annual temperature values using Sen method. | | World Bank | | 1991-2015 | | All (slope calculated) | | 227 | | (6) | |
|  |  | Change over time in total precipitation (mm) | | | Total precipitation (mm). Change over time estimated through linear slope estimation on annual precipitation values using Sen method. | | World Bank | | 1991-2015 | | All (slope calculated) | | 205 | | (7) | |
|  |  | Change over time in temperature variability (degree Celsius) | | | Temperature variability (degree Celsius). Change over time estimated through linear slope estimation of increase in annuals standard deviations using Sen method. | | World Bank | | 1991-2015 | | All (slope calculated) | | 227 | | (8) | |
|  |  | Change over time in precipitation variability (mm) | | | Precipitation variability (mm). Change over time calculated through linear slope estimation of increase in annuals standard deviations using Sen method. | | World Bank | | 1991-2015 | | All (slope calculated) | | 205 | | (9) | |
|  | Intensification of the agricultural sector | Change over time in cereal yield (kg per hectare) | | | Cereal yield, measured as kilograms per hectare of harvested land, includes wheat, rice, maize, barley, oats, rye, millet, sorghum, buckwheat, and mixed grains. Production data on cereals relate to crops harvested for dry grain only. Cereal crops harvested for hay or harvested green for food, feed, or silage and those used for grazing are excluded. The FAO allocates production data to the calendar year in which the bulk of the harvest took place. Most of a crop harvested near the end of a year will be used in the following year. Change over time estimated through the difference in medians calculated for the periods 2004/2006 and 2014/2016. | | World Bank | | 1961-2016 | | 2004-2016 (2015-2005, median difference) | | 179 | | (10) | |
|  |  | Change over time in agricultural area (% of land area) | | | Agricultural land refers to the share of land area that is arable, under permanent crops, and under permanent pastures. Arable land includes land defined by the FAO as land under temporary crops (double-cropped areas are counted once), temporary meadows for mowing or for pasture, land under market or kitchen gardens, and land temporarily fallow. Land abandoned as a result of shifting cultivation is excluded. Land under permanent crops is land cultivated with crops that occupy the land for long periods and need not be replanted after each harvest, such as cocoa, coffee, and rubber. This category includes land under flowering shrubs, fruit trees, nut trees, and vines, but excludes land under trees grown for wood or timber. Permanent pasture is land used for five or more years for forage, including natural and cultivated crops. Change over time estimated through the difference in medians calculated for the periods 2003/2005 and 2014/2016. | | World Bank | | 1961-2015 | | 2003-2015 (2014-2004, median difference) | | 206 | | (11) | |
|  | Improved access to infrastructure and information | | Change in access to infrastructure and information (electrical change) | The Defense Meteorological Program (DMSP) Operational Line-Scan System (OLS) has a unique capability to detect visible and near-infrared (VNIR) emission sources at night. This collection contains global nighttime lights images with no sensor saturation. The sensor is typically operated at a high-gain setting to enable the detection of moonlit clouds. However, with six bit quantization and limited dynamic range, the recorded data are saturated in the bright cores of urban centers. A limited set of observations at low lunar illumination were obtained where the gain of the detector was set significantly lower than its typical operational setting (sometimes by a factor of 100). Sparse data acquired at low-gain settings were combined with the operational data acquired at high-gain settings to produce the set of global nighttime lights images with no sensor saturation. Data from different satellites were merged and blended into the final product in order to gain maximum coverage. Change over time estimated through the difference in values between 2008 and 2015. | | NOAA (DMSP OLS: Global Radiance-Calibrated Nighttime Lights Version 4, Defense Meteorological Program Operational Linescan System) | | 2008-2015 | | 2015-2008 | | 246 | | (12) | |  |
|  | Soil degradation | | Land degradation (GLASOD degrees) | Erosion is the process by which soil is removed from a certain region due to the action of natural factors (wind, water, ice), of living organisms, and of gravity. Erosion is a natural process, but human activities can greatly influence its rate, especially through agriculture and deforestation. According to the U.N. Millennium Ecosystem Assessment, approximately 40% of the world's agricultural land is seriously degraded. In natural conditions, only very severe meteorological events will cause erosion, as the vegetation cover, the leaf litter and the organic matter will protect the soil absorbing rain impacts and preventing soil removal. Removal of the natural vegetation cover due to practices such as: deforestation; overgrazing; or industrial farming practices (e.g. tillage), leaves the soil exposed to the action of climatic factors, such as rain and wind. 1991 value used as the estimation of the level of land degradation. | | FAOSTAT data | | 1991 | |  | | 180 | | (13) | |  |
|  |  | | Soil erosion (GLASOD degrees) | Erosion is the process by which soil is removed from a certain region due to the action of natural factors (wind, water, ice), of living organisms, and of gravity. Erosion is a natural process, but human activities can greatly influence its rate, especially through agriculture and deforestation. According to the U.N. Millennium Ecosystem Assessment, approximately 40% of the world's agricultural land is seriously degraded. In natural conditions, only very severe meteorological events will cause erosion, as the vegetation cover, the leaf litter and the organic matter will protect the soil absorbing rain impacts and preventing soil removal. Removal of the natural vegetation cover due to practices such as: deforestation; overgrazing; or industrial farming practices (e.g. tillage), leaves the soil exposed to the action of climatic factors, such as rain and wind. 1991 value used as the estimation of the level of soil erosion. | | FAOSTAT data | | 1991 | |  | | 143 | | (14) | |  |
|  | Technological Innovation | | Change over time in ratio of cereal crop yield and fertilizer application | Cereal yield, measured as kilograms per hectare of harvested land, includes wheat, rice, maize, barley, oats, rye, millet, sorghum, buckwheat, and mixed grains. Production data on cereals relate to crops harvested for dry grain only. Cereal crops harvested for hay or harvested green for food, feed, or silage and those used for grazing are excluded. The FAO allocates production data to the calendar year in which the bulk of the harvest took place. Most of a crop harvested near the end of a year will be used in the following year. Fertilizer consumption measures the quantity of plant nutrients used per unit of arable land. Fertilizer products cover nitrogenous, potash, and phosphate fertilizers (including ground rock phosphate). Traditional nutrients--animal and plant manures--are not included. For the purpose of data dissemination, FAO has adopted the concept of a calendar year (January to December). Some countries compile fertilizer data on a calendar year basis, while others are on a split-year basis. Arable land includes land defined by the FAO as land under temporary crops (double-cropped areas are counted once), temporary meadows for mowing or for pasture, land under market or kitchen gardens, and land temporarily fallow. Land abandoned as a result of shifting cultivation is excluded. Change over time estimated through the difference in medians calculated for the periods 2001/2003 and 2012/2014. | | World Bank | | 2002-2014 | | 2002-2013 (2012-2003, median difference) | | 149 | | (15) | |  |
|  |  | | Change over time in fertilizer consumption (kg/ha of arable land) | Fertilizer consumption measures the quantity of plant nutrients used per unit of arable land. Fertilizer products cover nitrogenous, potash, and phosphate fertilizers (including ground rock phosphate). Traditional nutrients--animal and plant manures--are not included. For the purpose of data dissemination, FAO has adopted the concept of a calendar year (January to December). Some countries compile fertilizer data on a calendar year basis, while others are on a split-year basis. Arable land includes land defined by the FAO as land under temporary crops (double-cropped areas are counted once), temporary meadows for mowing or for pasture, land under market or kitchen gardens, and land temporarily fallow. Land abandoned as a result of shifting cultivation is excluded. Change over time estimated as the difference in medians calculated for the periods 2001/2003 and 2013/2015. | | World Bank | | 2002-2014 | | 2002-2014 (2013-2003, median difference) | | 152 | | (16) | |  |
| Trade/ Distribution | Trade policies to facilitate or mitigate trade expansion | | Change over time in food exports (% of merchandise exports) | Food comprises the commodities in SITC sections 0 (food and live animals), 1 (beverages and tobacco), and 4 (animal and vegetable oils and fats) and SITC division 22 (oil seeds, oil nuts, and oil kernels). Change over time estimated as the difference in medians calculated for the periods 2003/2005 and 2014/2016. | | World Bank | | 2000-2015 | | 2004-2016 (2015-2005, median difference) | | 144 | | (17) | |  |
|  | Internationalization of private investments | | Change over time in foreign direct investment (US$ dollars per capita) | Foreign direct investment refers to direct investment equity flows in the reporting economy. It is the sum of equity capital, reinvestment of earnings, and other capital. Direct investment is a category of cross-border investment associated with a resident in one economy having control or a significant degree of influence on the management of an enterprise that is resident in another economy. Ownership of 10 percent or more of the ordinary shares of voting stock is the criterion for determining the existence of a direct investment relationship. Data are in current U.S. dollars. Change over time estimated as the difference in medians calculated for the periods 2003/2005 and 2014/2016. | | World Bank | | 2000-2015 | | 2004-2016 (2015-2005, median difference) | | 196 | | (18) | |  |
|  |  | | Change over time in merchandise and services trade (US$ dollars per capita) | Services refer to economic output of intangible commodities that may be produced, transferred, and consumed at the same time. Data are in current U.S. dollars. Merchandise imports show the c.i.f. value of goods received from the rest of the world valued in current U.S. dollars. Merchandise exports show the f.o.b. value of goods provided to the rest of the world valued in current U.S. dollars. Change over time estimated as the difference in medians calculated for the periods 2003/2005 and 2014/2016. | | World Bank | | 2000-2015 | | 2004-2016 (2015-2005, median difference) | | 197 | | (19) | |  |
|  | Growing concerns for food safety | | Change over time of interest in aggregate of unsafe food, food quality control, and traceability | Interest in food safety assessed through Google Trend® data between 2004 and 2018 (adjusted per capita) using the aggregated counts for the search of the following key-words: "unsafe food", "food quality control", and "traceability" in eight different languages: English, French, Spanish, Portuguese, German, Dutch and Russian. Change over time calculated through the estimation of the linear slope using Sen method. | | Google Trends | | 2004-2019 | | Available data | | 69 | | (20) | |  |

Notes URL

(1) https://trends.google.com/trends/?geo=US

(2) https://data.worldbank.org/indicator/SP.POP.GROW

(3) https://data.worldbank.org/indicator/ny.gdp.mktp.kd.zg

(4) https://data.worldbank.org/indicator/sp.urb.totl.in.zs

(5) https://data.worldbank.org/indicator/sl.srv.empl.fe.zs

(6) https://climateknowledgeportal.worldbank.org/download-data

(7) https://climateknowledgeportal.worldbank.org/download-data

(8) https://climateknowledgeportal.worldbank.org/download-data

(9) https://climateknowledgeportal.worldbank.org/download-data

(10) https://data.worldbank.org/indicator/AG.YLD.CREL.KG?view=chart

(11) https://data.worldbank.org/indicator/ag.lnd.agri.zs

(12) https://ngdc.noaa.gov/eog/dmsp/download_radcal.html

(13) http://www.fao.org/faostat/en/#data/ES

(14) http://www.fao.org/faostat/en/#data/ES

(15) https://data.worldbank.org/indicator/AG.YLD.CREL.KG?view=chart

(16) https://data.worldbank.org/indicator/ag.con.fert.zs

(17) https://data.worldbank.org/indicator/tx.val.food.zs.un

(18) https://data.worldbank.org/indicator/BX.KLT.DINV.CD.WD?locations=PE

(19) https://data.worldbank.org/indicator

(20) https://trends.google.com/trends/?geo=US
