# Supplementary material for: Global drivers of food system (un)sustainability: A multi-country correlation analysis
Source: PLoS One. 2020 Apr 3;15(4):e0231071. doi: 10.1371/journal.pone.0231071 (PMC7122815; doi:10.1371/journal.pone.0231071)
Supplement: S1 Fig — (DOCX) [file pone.0231071.s006.docx]

S1 Figure. PRISMA flow diagram describing the selection process regarding the indicators to be included in the computation of the final food system sustainability score.
