# Supplementary material for: Global drivers of food system (un)sustainability: A multi-country correlation analysis
Source: PLoS One. 2020 Apr 3;15(4):e0231071. doi: 10.1371/journal.pone.0231071 (PMC7122815; doi:10.1371/journal.pone.0231071)
Supplement: S2 Fig — High positive correlations are indicated in dark blue, while high negative correlations are showed in dark red. The diagram shows that the environmental and social dimensions are characterized by low internal cross-correlations, while the economic and food & nutrition dimensions display a larger number of high positive and/or negative cross-correlations. (DOCX) [file pone.0231071.s007.docx]

S2 Figure. Spearman-correlation matrix of the 27 indicators included in the food system sustainability metric. High positive correlations are indicated in dark blue, while high negative correlations are showed in dark red. The diagram shows that the environmental and social dimensions are characterized by low internal cross-correlations, while the economic and food & nutrition dimensions display a larger number of high positive and/or negative cross-correlations.
