# Supplementary material for: Global drivers of food system (un)sustainability: A multi-country correlation analysis
Source: PLoS One. 2020 Apr 3;15(4):e0231071. doi: 10.1371/journal.pone.0231071 (PMC7122815; doi:10.1371/journal.pone.0231071)
Supplement: S3 Fig — The frontier shows that the larger the number of indicators considered, the smaller the number of countries for which those indicator datasets are complete, and vice versa. (DOCX) [file pone.0231071.s008.docx]

S3 Figure. Trade-off ‘frontier’ between the number of countries for which the indicator datasets are complete and the number of indicators included in the metric. The frontier shows that the larger the number of indicators considered, the smaller the number of countries for which those indicator datasets are complete, and vice versa.
