# Supplementary material for: Global drivers of food system (un)sustainability: A multi-country correlation analysis
Source: PLoS One. 2020 Apr 3;15(4):e0231071. doi: 10.1371/journal.pone.0231071 (PMC7122815; doi:10.1371/journal.pone.0231071)
Supplement: S4 Fig — High positive correlations are indicated in dark blue, while high negative correlations are showed in dark red. The diagram shows that very little cross-correlations occur. (DOCX) [file pone.0231071.s009.docx]

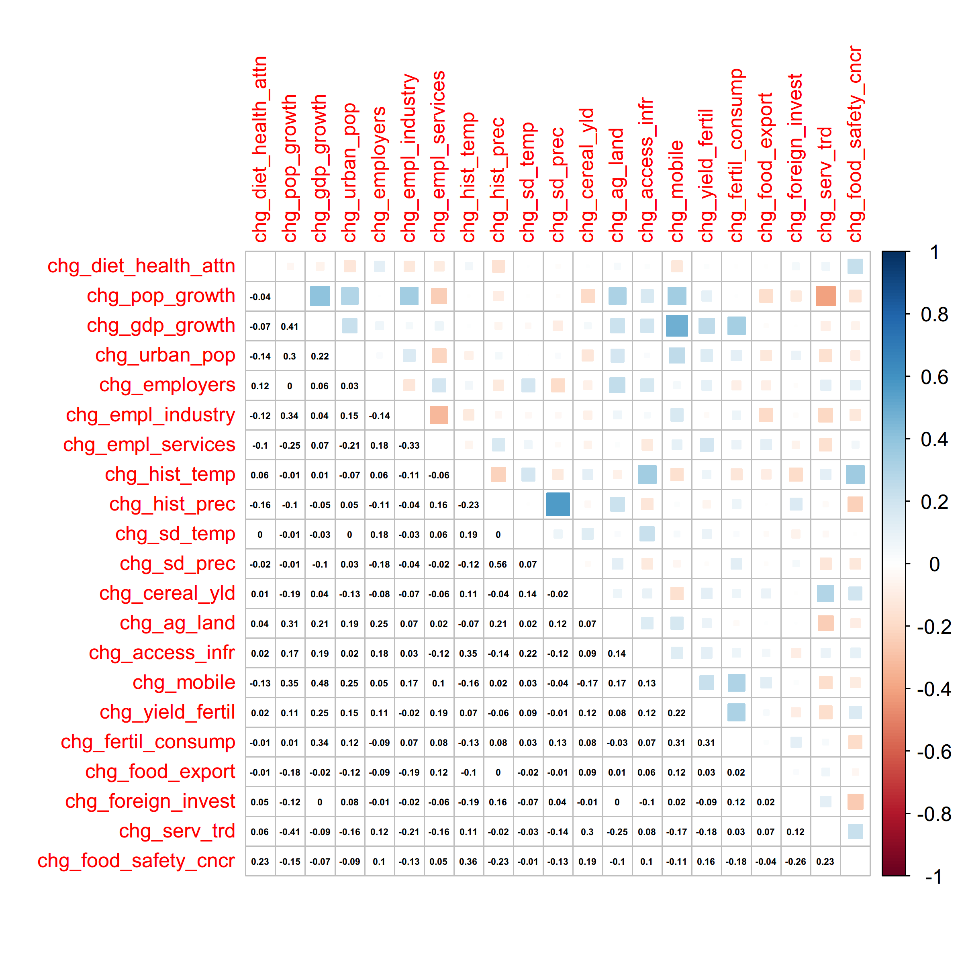


S4 Figure. Cross-correlation matrix of the 20 drivers’ indicators. High positive correlations are indicated in dark blue, while high negative correlations are showed in dark red. The diagram shows that very few cross-correlations are observed between the 20 variables.
